# Early infection response of fungal biotroph *Ustilago maydis* in maize

**DOI:** 10.3389/fpls.2022.970897

**Published:** 2022-09-09

**Authors:** Kunkun Zou, Yang Li, Wenjie Zhang, Yunfeng Jia, Yang Wang, Yuting Ma, Xiangling Lv, Yuanhu Xuan, Wanli Du

**Affiliations:** ^1^College of Agronomy, Shenyang Agricultural University, Shenyang, China; ^2^College of Agronomy, Heilongjiang Bayi Agricultural University, Daqing, China; ^3^College of Plant Protection, Shenyang Agricultural University, Shenyang, China

**Keywords:** chloroplast, sugar transport, transcriptome, *Ustilago maydis*, *Zea mays* L.

## Abstract

Common smut, caused by *Ustilago maydis* (DC.) Corda, is a destructive fungal disease of maize worldwide; it forms large tumors, reducing corn yield and quality. However, the molecular defense mechanism to common smut in maize remains unclear. The present study aimed to use a leading maize inbred line Ye478 to analyze the response to *U. maydis* inoculation. The histological and cytological analyses demonstrated that *U. maydis* grew gradually to the host cells 6 h post-inoculation (hpi). The samples collected at 0, 3, 6, and 12 hpi were analyzed to assess the maize transcriptomic changes in response to *U. maydis*. The results revealed differences in hormone signaling, glycometabolism, and photosynthesis after *U. maydis* infection; specific changes were detected in jasmonic acid (JA), salicylic acid (SA), ethylene (ET), and abscisic acid (ABA) signaling pathways, glycolysis/gluconeogenesis, and photosystems I and II, probably related to defense response. MapMan analysis demonstrated that the differentially expressed genes between the treatment and control groups were clustered into light reaction and photorespiration pathways. In addition, *U. maydis* inoculation induced chloroplast swelling and damage, suggesting a significant effect on the chloroplast activity and subsequent metabolic process, especially hexose metabolism. A further genetic study using wild-type and galactinol-sucrose galactosyltransferase (*gsg*) and yellow-green leaf-1 (*ygl-1*) mutants identified that these two *U. maydis*-induced genes negatively regulated defense against common smut in maize. Our measurements showed the pathogen early-invasion process, and the key pathways of both chlorophyll biosynthesis and sugar transportation were critical modified in the infected maize line, thereby throwing a light on the molecular mechanisms in the maize-*U. maydis* interaction.

## Introduction

Common smut is a plant disease caused by the fungus *Ustilago maydis* (DC.) Corda., belonging to the Basidiomycetes subphylum (class Ustilaginales, family Ustilaginaceae, and genus *Ustilago*). It is a facultative biotrophic fungus that depends on sexual reproduction to cause disease (Wahl et al., 2010). The basidiomycete *U. maydis* is a major pathogen that infects maize crops worldwide, influencing the quantity and quality of corn (Martinez-Espinoza et al., 2002). The pathogen has developed a highly specialized adaptation system with the host plant that allows it to infect all aerial parts of maize, causing tumors in the tender tissues. The fungal hyphae traverse plant cells, grow intercellularly, and establish an intimate interaction, inhibiting the host defense by hindering nutrient supply (Skibbe et al., 2010; Villajuana-Bonequi et al., 2019). It is a dimorphic fungus that infects the host plant during the dikaryotic stage (Zuo et al., 2019). Initially, *U. maydis* grows along the epidermis cell of the host plant and forms a specialized infection structure called appressorium that further penetrates the epidermal cells *via* the action of cell wall-degrading enzymes. Finally, it forms tumors, releasing diploid teliospores when mature (15 days post-infection, dpi; Walbot and Skibbe, 2010; Ferris and Walbot, 2021). Additionally, *U. maydis* is a typical model organism for studying biotrophic plant-pathogen interactions (Schmitz et al., 2020).

Many defense components, such as carbohydrates, enzymes, phytohormones, and signaling molecules, are activated during pathogen attacks, which firmly cooperate to build a fine system that can optimize growth adaptation (Schurack, 2021). For initial penetration and intracellular growth, *U. maydis* degrades the cell wall, which acts as the first plant defense line during pathogen invasion. Furthermore, the secondary wall lignification restricts the tumor formation induced by *U. maydis* (Doehlemann et al., 2008b; Matei et al., 2018). Therefore, the fungus has to overcome the improved reinforcement of cell wall components to complete infection. Studies showed that several genes involved in the process of photosynthesis were strongly enriched during *U. maydis*-induced host responses and upregulated in resistant maize lines (Schurack, 2021). Meanwhile, studies also showed a decrease in the expression of the genes related to photosynthesis in *U. maydis*-infected tissue, with impaired chloroplast and photosynthetic functions and disturbed source-sink relationship (Doehlemann et al., 2008a). The correlation between photosynthesis and *U. maydis* revealed that the resistant maize performed photosynthesis more efficiently, changed energy re-allocation, and decreased fungal proliferation, subsequently leading to disease resistance. Sugar transport and partitioning also played key roles in regulating resistance to common smut in maize. However, the pathogens have evolved opposing mechanisms with host plants (Sosso et al., 2015, 2019). Wahl et al. identified a high-affinity sucrose symporter Srt1 in the fungus *U. maydis*, which was located on the plasma membrane of the invasive hyphae (Wahl et al., 2010). It competed with the plant SUC (or SUT) sucrose transporters and invertases for the apoplastic sucrose, resulting in glucose and fructose production (Sauer et al., 2004; Carpaneto et al., 2005). Meanwhile, Matei et al. found high starch accumulation in the tumorous tissues of the infected leaves (Matei et al., 2018). During tumor formation, the fungus stimulated maize growth by deploying its own sugar transporters, which caused a sugar gradient within the fungus and then increased Glc levels in tips (Sosso et al., 2019). In addition, *U. maydis* triggers local sugar transporter genes *ZmSWEET4a* and *ZmSWEET4b*, which subsequently recruit the SWEET transporters to seep sugars into the apoplast at the biotrophic interface. Studies also proved a significant increase in free sucrose and hexose accumulation in infected leaves, which served as an easily accessible carbon source for the fungus (Doehlemann et al., 2008a; Horst et al., 2010b). However, the molecular regulation of sugar partitioning between *U. maydis* and the host at the interface during tumor development is not clear.

Generally, effectors are perceived as small proteins secreted by the pathogen; these molecules are crucial factors for *U. maydis* colonization. The effector Pep1 inhibits pattern-triggered reactive oxygen species (ROS) bursts and suppresses maize peroxidase POX12, which is essential for the successful penetration at the early stage of infection (Doehlemann et al., 2009; Hemetsberger et al., 2012). Meanwhile, effector Pit2 inhibits a set of apoplastic cysteine proteases of maize through a conserved microbial motif to prevent salicylic acid (SA)-associated plant defenses in the early stages of the infection (Mueller et al., 2013). The *U. maydis* virulence promotes the secretion of the effector protein Tin2 that interacts with the maize protein kinase ZmTTK1 inside plant cells, redirecting the lignin biosynthesis pathway toward anthocyanin production in the infected tissues and vascular bundles (Tanaka et al., 2014). During growth, Rsp3 (repetitive secreted protein 3) decorates the hyphal surface and interacts with at least two secreted maize DUF26-domain family proteins, AFP1 and AFP2, whose silencing partially rescues the virulence function of the smut fungi (Ma et al., 2018). Additionally, the secreted fungalysin UmFly1 modulates both plant and fungal chitinases; it reduces the cleavage of the maize chitinase ZmChiA and promotes fungal infection (Ökmen et al., 2018). More recently, it was found that the secreted fungal chorismate mutase Cmu1 interacted with maize-encoded kiwellins (ZmKWL1), significantly inhibiting Cmu1 catalytic activity and further counteracting the pathogen virulence (Han et al., 2019). Overall, the studies indicated that *U. maydis* effectors were organ-specific during biotroph development and key determinants of *U. maydis* virulence.

Generally, a broad physiological reprogramming in maize occurs in response to *U. maydis* infection, including changes in various phytohormones, jasmonate (JA)-induced genes such as defensins and chitinases, and auxins and JA-signaling components (Wang et al., 2007). Furthermore, secondary metabolite synthesis is triggered, and cell division processes are upregulated, inducing pathogenesis (PR)-related genes (Doehlemann et al., 2008a,b; Lanver et al., 2018). Skibbe et al. (2010) investigated the changes in the expression patterns of genes regulating the interaction between *U. maydis* and maize 1–9 days after inoculation through RNA-sequencing (RNA-Seq). However, the transcriptome during the early response (3–12 h) to *U. maydis* infection was not reported.

Therefore, the present study aimed to elucidate the early response of maize infected with *U. maydis*. The cellular morphology and primary and secondary metabolism were analyzed in various stages of the infection to reveal the cell-specific changes during tumorigenesis. Further, RNA-Seq was performed using maize leaves inoculated with *U. maydis*, and the genes related to the regulation of maize defense to common smut were analyzed. The findings on the early response provided novel and valuable information for understanding the unique resistance mechanism during *U. maydis* infection.

## Materials and methods

### Plant growth and *U. maydis* inoculation

The common smut susceptible inbred line Ye478, the wild-type (WT) inbred line B73, and the galactinol-sucrose galactosyltransferase (*gsg*) (GeneID: *Zm00001d031303*) and yellow-green leaf-1 (*ygl-1*) (GeneID: *GRMZM2G007441*) mutants of maize (*Zea mays* L.) were used in this study. All plants were grown in an artificial climate chamber (*T* = 28°C, 14 h day/10 h night, and relative humidity = 50%) at the College of Agronomy, Shenyang Agricultural University, China, and infected with *U. maydis* (FB1 × FB2). An experiment was conducted in a completely randomized design using six seedlings per replicate and three replicates per line. For the artificial stab inoculation of three-week-old maize seedlings, the *U. maydis* strain FB1 × FB2 was grown at 28°C in yeast extract peptone and sucrose light (YEPSL; 0.4% yeast extract, 0.4% peptone, and 2% sucrose) as described in an earlier study (Matei et al., 2018). Then, three-week-old seedlings were inoculated with *U. maydis*, and their leaves were sampled after 0, 3, 6, and 12 hpi to analyze the *U. maydis* infection-mediated differences in sheath cells, metabolites, and gene expression.

### Microscopic analysis

The fungal hyphae in the infected leaf tissues were harvested and cleared in the Carnot fixator overnight at room temperature. The samples were then rinsed and stained with WGA-Alexa Fluor 488 (Invitrogen, CA, USA), and the plant membrane was visualized using propidium iodide (PI), as described in an earlier study (Gao et al., 2013). The samples were then observed under a confocal microscope (Zeiss, LSM 780, Germany).

Scanning electron microscopy (SEM) was carried out to observe the infection dynamics of samples collected at different time points after inoculation. The samples were fixed in glutaraldehyde for 2 h and continuously dehydrated with alcohol and *tert*-butanol, followed by freeze-drying (Vacuum Device, VFD-30, Japan). These samples were coated with gold powder by spraying (Hitachi, MSP-2S, Japan) and observed using an SEM (Hitachi, TM-3030, Japan).

Furthermore, the leaf samples were analyzed by transmission electron microscopy (TEM) to determine the subcellular structural differences. The samples were fixed overnight in glutaraldehyde and dehydrated in acetone. These light microscopy and electron microscopy samples were processed as described by Redkar et al. (2015). Ultrathin sections were made using a Leica ultramicrotome (Leica, EM UC7, Germany) and observed under a Hitachi TEM (Hitachi, HT7700, Japan).

### Primary and secondary metabolite analysis

The leaf samples were collected 0, 3, 6, 12, and 24 h after FB1 × FB2 inoculation, using ddH_2_O as control. The inoculation was carried out at the same leaf position for each plant; samples were collected from six plants per treatment and per time point to obtain the mixed samples, immediately frozen in liquid nitrogen, and stored at −80°C. These samples were used to extract the supernatant with the extraction kit (Suzhou Keming Bioengineering Institute, Jiangsu, China). ROS, such as superoxide anion (O_2•_^−^), hydrogen peroxide (H_2_O_2_), and hydroxyl radical (OH_•_), in these samples were quantified by spectrophotometric analysis (UV-2550, Shimadzu Corporation, Kyoto, Japan).

Furthermore, the accumulated phenylalanine ammonia-lyase (PAL), glutathione S-transferase (GST) (reduced glutathione, GSH; oxidized glutathione, GSSG), reduced ascorbic acid (ASA), and dehydroascorbic acid (DHA) in the leaf samples were extracted after inoculation and analyzed using specific extraction kits (Suzhou Keming Bioengineering Institute, Jiangsu, China); they were associated with maize defense response to common smut fungus. These stress metabolites were quantified using an automatic microplate reader (Thermo Scientific Multiskan FC, 1510, Vantaa, Finland).

Additionally, SA, 1-amino cyclopropyl-1-carboxylic acid (ACC, ethylene precursor), jasmonates (JA), and jasmonoyl–isoleucine (JA-ILE) play significant roles in plant responses to biotic stresses. SA, ACC, JA, and JA-ILE were extracted and purified as described in an earlier study (Bari and Jones, 2009). The phytohormone levels in the infected tissues were quantified by high-performance liquid chromatography–electrospray ionization tandem mass spectrometry (HPLC-ESI MS/MS, 6500 plus, MA, USA), using the standards supplied by Sigma (MO, USA). The contents were analyzed using GraphPad Prism 9.0 software.

### RNA extraction and qRT-PCR analysis

The leaf samples of 3-week-old maize seedlings inoculated with *U. maydis* were collected after 0, 3, 6, and 12 h, and RNA was isolated using the TRIzol reagent (Invitrogen, CA, USA). The cDNA was synthesized using the reverse transcription kit (TaKaRa). Finally, the gene expression levels were analyzed by quantitative reverse transcriptase–polymerase chain reaction (qRT-PCR) on a Bio-Rad CFX96 real-time PCR system (Bio-Rad, CA, USA) and quantified using the 2^−ΔΔ*Ct*^ method. The primers used are described in Supplementary Table 1. Excel 2022 and GraphPad Prism 9.0 were used to draw the figures.

### Sequencing and data analysis

Eighteen samples from different time points, including three biological replicates per time point, were sequenced on an Illumina platform (HiSeq 2000). The clean reads were aligned to the maize B73 reference genome (Zea_mays.B73_RefGen_v4) using the Tophat 2.0.7 software, a fast splice junction mapper for RNA-Seq reads. The clean reads were then counted using HTSeq0.6.1 and standardized as fragments per kilobase of exon model per million mapped reads (FPKM) (Anders et al., 2015). DESeq R package was used to analyze the genes differentially expressed between the treatments and the control (Anders and Huber, 2010); genes with an adjusted *P*-value < 0.05 were defined as differentially expressed genes (DEGs). These DEGs were further used for the Gene Ontology (GO) enrichment analysis by GOseq R package, where gene length bias was adjusted and a *P*-value < 0.05 was considered significant (Young et al., 2010). The Kyoto Encyclopedia of Genes and Genomes (KEGG) pathway analysis of DEGs was performed using the Cluster Profiler R package to identify the significantly enriched pathways; a *P*-value < 0.05 was used as the threshold of significance (Yu et al., 2012). The enriched pathways or processes were visualized, and a schematic diagram of DEGs was generated using the MapMan tool.

## Results

### *Ustilago maydis* hyphae proliferated in the host at the early infection phase

**Figure 1:**
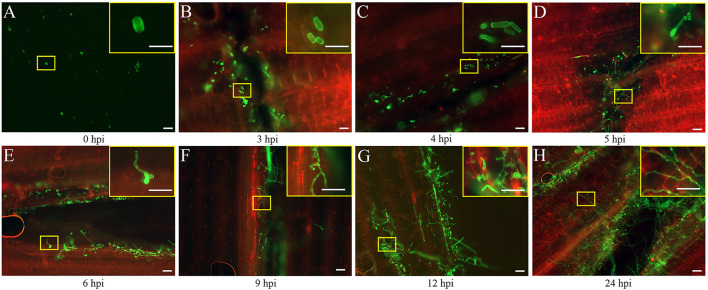
Early developmental characteristics of biotrophic *Ustilago maydis* in maize. **(A–H)** Maize leaves were collected at 0, 3, 4, 5, 6, 9, 12, and 24 hpi respectively with *U. maydis* FB1 × FB2 strain. The image with the yellow border in the upper right-hand corner represents a magnified view of the position of the yellow box. The development of the fungal hyphae was visualized *via* WGA-AF488 (green) staining. The plant cell wall was stained with propidium iodide (red) and observed using a confocal microscope. Scale bar = 50 μm.

**Figure 2:**
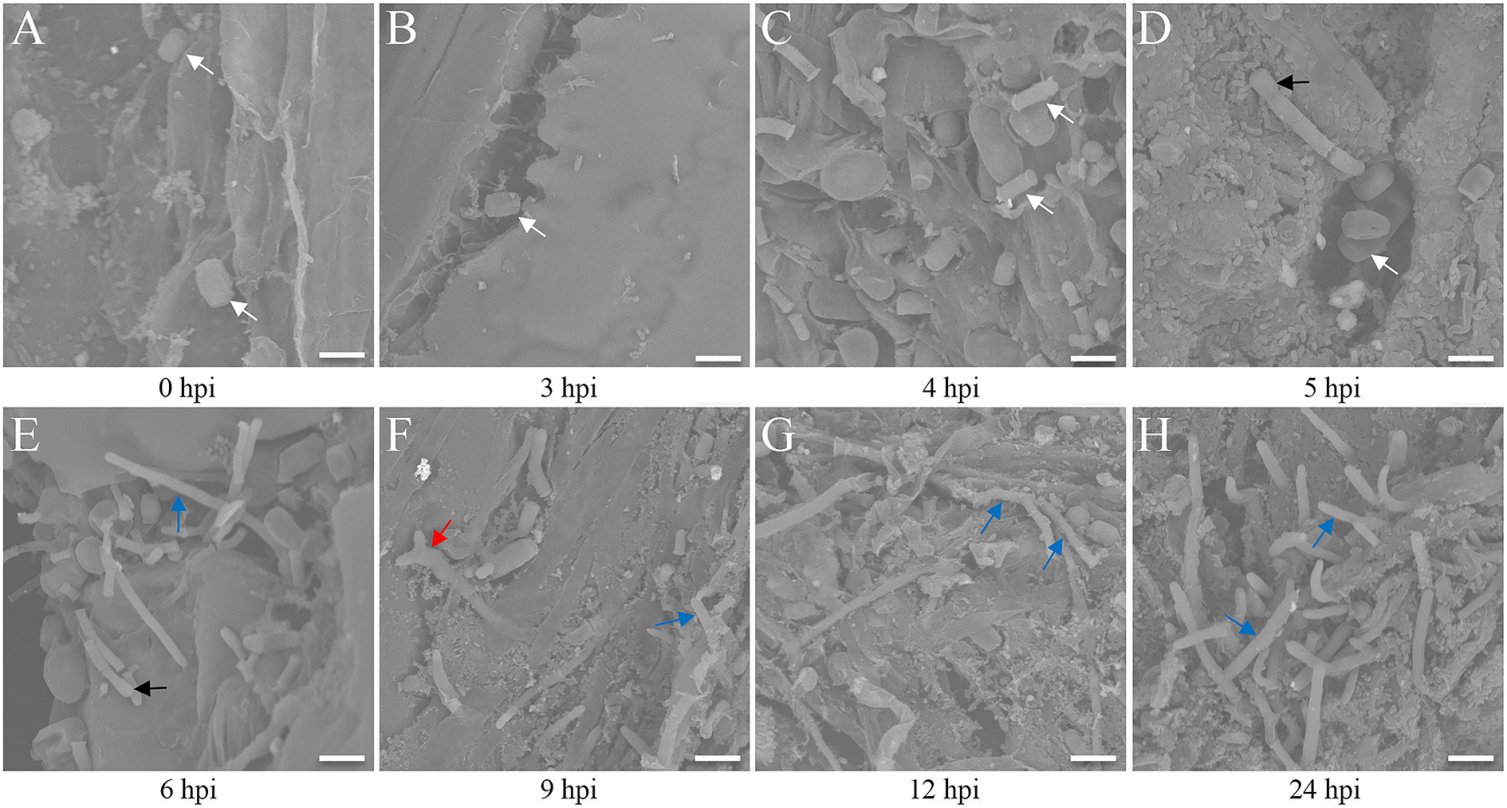
Scanning electron microscopy analysis of maize leaves after *Ustilago maydis* inoculation. **(A–H)** Three-week-old Ye478 seedlings cultivated in an artificial climate chamber were inoculated, and the hyphal developmental behavior on the interface was captured at 0, 3, 4, 5, 6, 9, 12, and 24 hpi, respectively. White arrow: strain; black arrow: appressorium; blue arrow: hypha; red arrow: branch. Scale bar = 100 μm.

The seedlings infected by the FB1 × FB2 strain were analyzed using confocal imaging to identify the earliest time point and investigate the maize leaf response to infection. The fungal growth within the leaf tissue at 0, 3, 4, 5, 6, 9, 12, and 24 hpi was visualized using the WGA-AF488/PI co-staining (Figure 1). Only a few FB1 × FB2 cells had divided and elongated at 5 hpi compared with those at 0, 3, and 4 hpi (Figures 1A–D). This study first observed filaments and appressoria during the infection process at 6 hpi (Figure 1E). At 9 hpi, the hyphae were closely found with short branched parts (Figure 1F). The fungus extended the hyphae to the proliferated, older parts, suggesting the initiation of plant defense. By 12 hpi, the hyphae branched and occupied the meristematic tissue, with intracellular and intercellular growth (Figure 1G). At 24 hpi, the hyphae profusely proliferated, with widespread transcellular infection in maize (Figure 1H). The microscopic images further revealed the hyphal development and plant cell wall reactions in various stages. Further, the sporidial features were analyzed 0, 3, 4, 5, 6, 9, 12, and 24 h after FB1 × FB2 inoculation using SEM (Figure 2). The analysis revealed consistent and rapid sporidial division and differentiation in the incisions with artificial acupuncture infection. By 3 hpi, sporidia on the leaf surface were morphologically similar to those in YEPSL (Figure 2B). By 4 hpi, the cells began to divide and further elongate (Figure 2C). The hyphae formed appressoria and penetrated the host cells as early as at 5 hpi, establishing special biotrophic interaction between the pathogen and the plant cells (Figure 2D). The hyphae proliferated rapidly and branched from 6 hpi, penetrated the cell wall, and caused intracellular and intercellular infections (Figures 2E,F). Further, as the infection progressed, the hyphae multiplied by overcoming the host defense system (Figures 2G,H).

### Genes identified during the response to *U. maydis* infection

**Figure 3:**
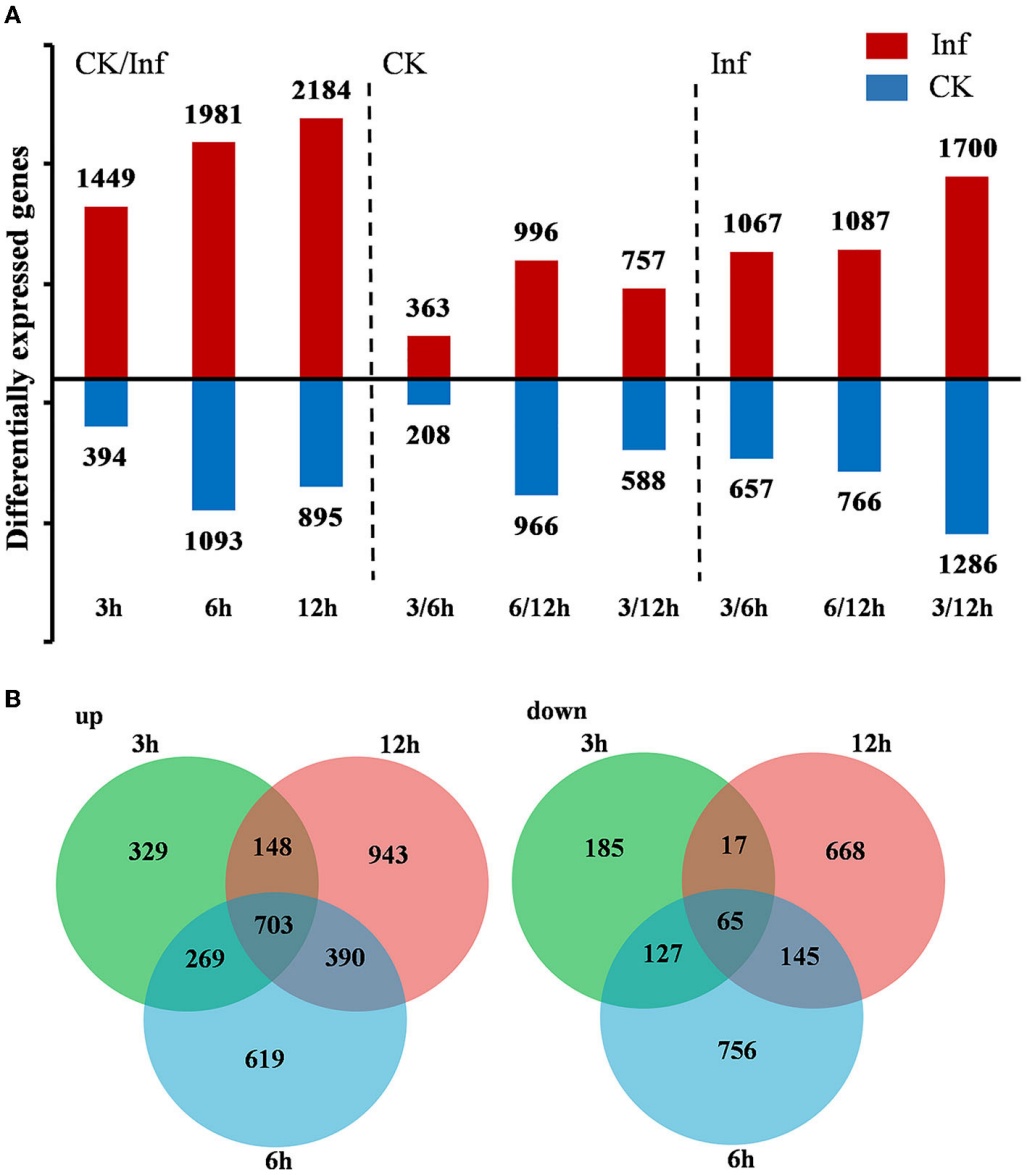
Differentially expressed genes (DEGs) between the control (CK) and *Ustilago maydis* inoculated samples after 3, 6, and 12 h, respectively. **(A)** Number of upregulated and downregulated genes after 3, 6, and 12 h in control vs. infected samples. **(B)** Venn diagram showing the unique and shared DEGs in the treatment groups at 3, 6, and 12 h, respectively. Plants infected with *U. maydis* (Inf) were analyzed at 3, 6, and 12 hpi respectively, using those injected with distilled water as control (CK).

DEGs were identified by comparing the gene expression profiles at 3, 6, and 12 h after *U. maydis* inoculation to investigate the transcriptome changes of Ye478 using samples injected with distilled water collected after 0 h as control. A total of 7,996 DEGs were identified, including 1,843, 3,074, and 3,079 after 3, 6, and 12 h, respectively; the numbers of upregulated and downregulated genes were compared. During *U. maydis* infection, the upregulated genes were more than downregulated at all three infection stages. Interestingly, the number of DEGs at 3 and 6 hpi was much less than that at 12 hpi, reflecting a slight change in transcriptome during the early phase of infection (3–6 hpi) (Figure 3A and Supplementary Table 2). In addition, the Venn diagram showed that 703 upregulated and 65 downregulated genes were shared among the three stages (Figure 3B). Most DEGs were different among the various infection stages, which indicated a complex plant response to infection.

**Figure 4:**
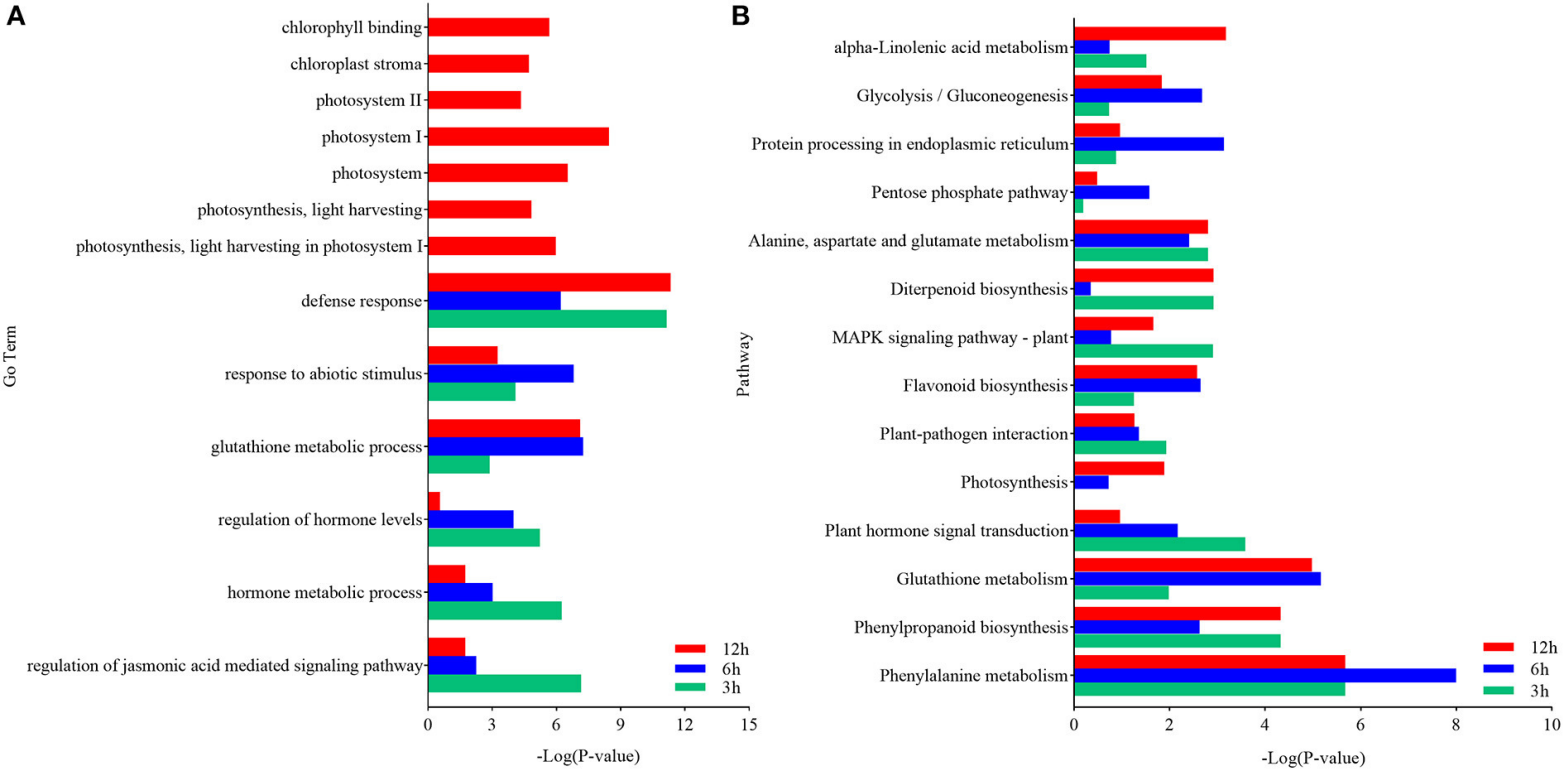
Functional analysis of DEGs in maize inoculated with *Ustilago maydis*. **(A)** GO enrichment analysis was executed with DEGs identified between control (CK) and inoculated samples (Inf). The ordinate and abscissa represent the main biological process GO terms and -Log (*P*-value), respectively. **(B)** Pathways significantly enriched with the DEGs identified between CK and Inf samples at the various time points after inoculation. The ordinate and abscissa represent the major KEGG biological pathways and -Log (*P*-value), respectively. The green bars represent CK 3 h vs. Inf 3 h; the blue bars represent CK 6 h vs. Inf 6 h; the red bars represent CK 12 h vs. Inf 12 h.

The DEGs in each pairwise comparison were further used for the GO enrichment analysis to identify the associated functions. The GO terms defense response (GO:0006952), response to abiotic stimulation (GO:0009628), glutathione metabolism process (GO:0006749), regulation of hormone levels (GO:0010817), and hormone metabolism (GO:0042445), especially JA-mediated signaling pathway (GO:2000022), were significantly enriched (Supplementary Figure 1 and Table 3). These terms indicated that defense response, glutathione metabolism, and JA signaling pathway were activated in response to *U. maydis* inoculation in Ye478 at the seedling stage. Moreover, photosystem I (GO:0009522), photosystem II (GO:0009523), photosynthesis, light harvesting in photosystem I (GO:0009768), chlorophyll-binding (GO:0016168), and chloroplast stroma (GO:0009570) were also significantly enriched (Figure 4 and Supplementary Table 3). These results indicated that the genes involved in the photosystem were quickly induced after *U. maydis* invasion, while the chloroplast-associated genes were firmly expressed and activated in the late stage of invasion.

KEGG analysis was performed to further clarify the biological functions of the DEGs. Compared with the CK3h vs. Inf3h, CK6h vs. Inf6h, and CK12h vs. Inf12h had 668 and 118 genes, respectively, that were significantly upregulated and downregulated at 3 time points. The most significantly enriched pathways during the defense response were plant-pathogen interaction, plant hormone signal, mitogen-activated protein kinase(MAPK) signaling pathway, glycolysis/gluconeogenesis, phenylpropanoid biosynthesis, protein processing in the endoplasmic reticulum, photosynthesis, cysteine and methionine metabolism, glutathione metabolism, and other processes (Figure 4 and Supplementary Figure 2, Supplementary Table 4). The integrated GO and KEGG enrichment analysis indicated that hormone signal transduction of SA, ethylene (ET), and JA, glycolysis, glycogenesis, and photosynthesis pathways played significant roles in *U. maydis* infection response.

**Figure 5:**
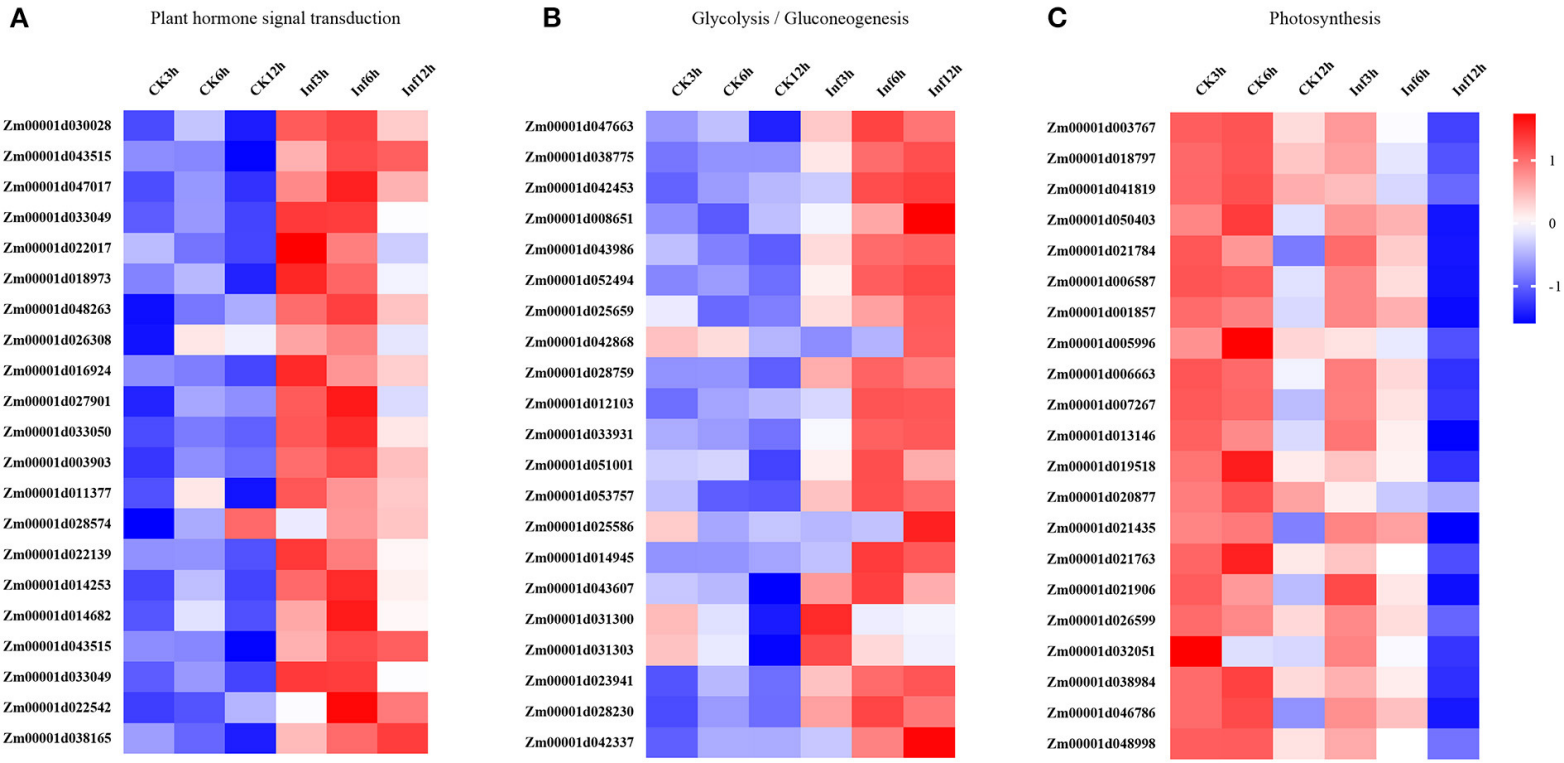
Changes in the expression levels of DEGs 3, 6, and 12 h, respectively after *Ustilago maydis* inoculation associated with KEGG pathways. Different colors represent differences in gene expression between CK and Inf samples (upregulated, red; downregulated, blue; mean, white). **(A)** Plant hormone signal transduction pathway-related genes. **(B)** Glycolysis/gluconeogenesis pathway-related genes. **(C)** Photosynthesis-related genes.

Additionally, the DEGs involved in hormone signaling, glycometabolism, and photosynthesis were investigated to analyze their vital roles in Ye478 disease resistance. The analysis of data related to the hormone signal transduction and pathogen identification showed that many genes involved in JA, SA, ET, or ABA signaling pathways were significantly enhanced in the Ye478 leaves after 3 h of *U. maydis* infection (Figure 5A). Then, a complex network constructed to understand the plant defense response showed that numerous polysaccharides or flagellin genes related to the pathogen-triggered immunity (PTI) mechanism of pathogen recognition were dramatically induced after 6 h of *U. maydis* inoculation, which subsequently led to glycometabolism, glycolysis/gluconeogenesis, and resistance gene expression (Figure 5B). Finally, the photosynthesis comprising the responsive genes of photosystems I and II was found to be one of the most significantly enriched pathways at 12 hpi (Figure 5C and Supplementary Table 5). These results suggested that a complex mechanism involving PTI that recognizes regulates plant defense against pathogen invasion.

**Figure 6:**
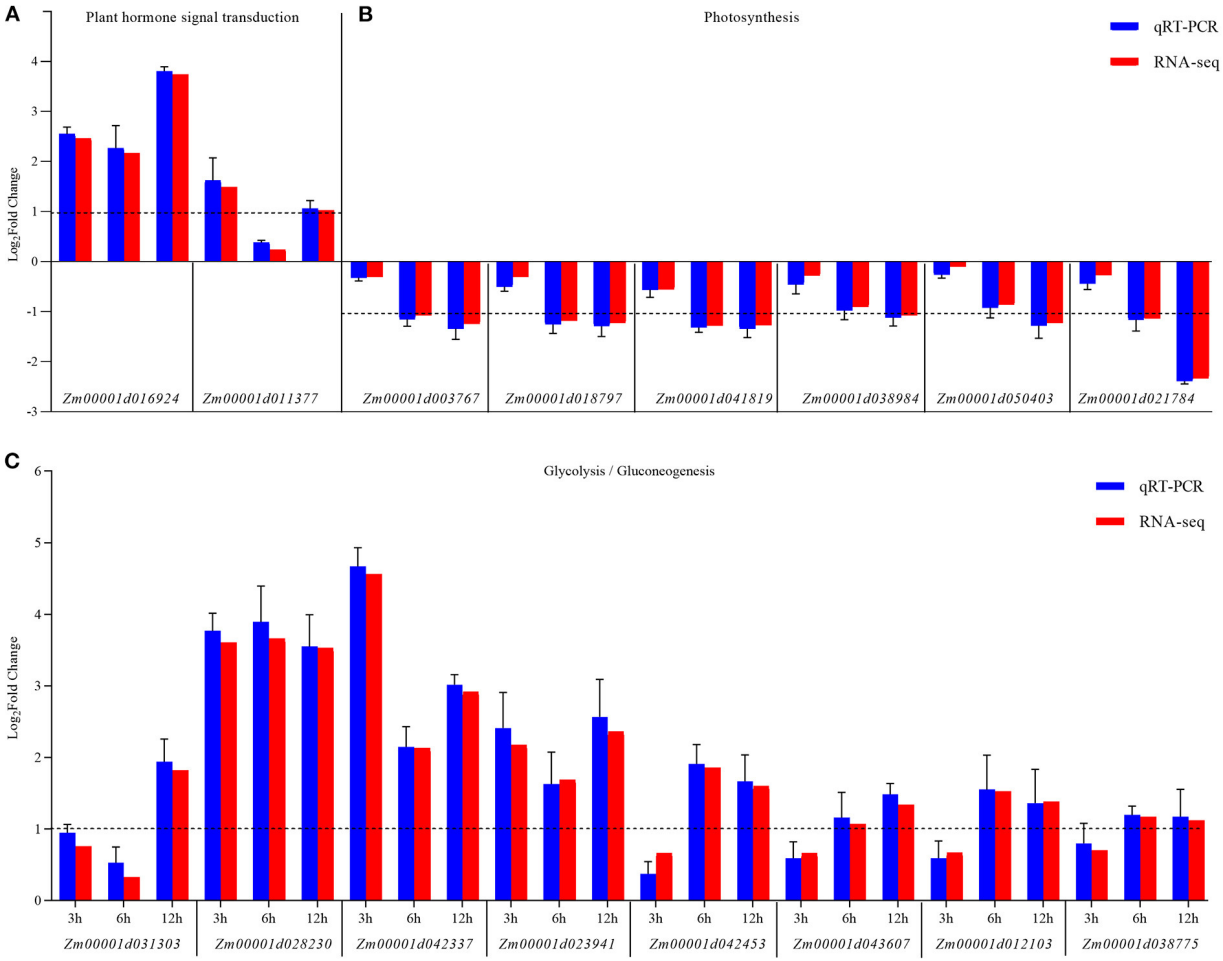
qRT-PCR validation of the expression of KEGG pathway-related genes differentially expressed between the control (CK) and *Ustilago maydis-*infected samples (Inf) at 3, 6, and 12 hpi, respectively. **(A)** Plant hormone signal transduction pathway-related genes. **(B)** Photosynthesis-related genes. **(C)** Glycolysis/gluconeogenesis-related genes. The blue bars represent the Log_2_-fold change in expression levels based on qRT-PCR, and the red bars represent the Log_2_-fold change in expression levels based on RNA-seq. Error bars indicate the standard deviation of three biological replicates. The dotted line is shown at a Log_2_-fold change of 1; genes with a fold change≥2 were identified as differentially expressed.

Furthermore, the changes in the expression levels of a few representative genes were analyzed by qRT-PCR to confirm the results. Sixteen genes were chosen for further analysis as follows: *Zm00001d016924* and *Zm00001d011377* related to hormone signal transduction; *Zm00001d01003767, Zm00001d018797, Zm00001d01041819, Zm00001d01038984, Zm00001d01050403*, and *Zm00001d021784* related to glycolysis/gluconeogenesis; *Zm00001d031303, Zm00001d028203, Zm00001d042337, Zm00001d01023941, Zm00001d01042453, Zm00001d01043607, Zm00001d01012103*, and *Zm00001d01038775* related to photosynthesis. The fold changes in the expression of these genes showed that the RNA-Seq data were consistent with the qRT-PCR results (Figure 6 and Supplementary Table 6).

Finally, an overview of the metabolic pathways in response to *U. maydis* inoculation was mapped using MapMan. The analysis revealed significant enrichment of genes regulating secondary metabolites at 3 hpi, followed by hormone signal transduction and plant–pathogen interaction (Supplementary Figure 3A). Phenylpropanoid biosynthesis, glycolysis/gluconeogenesis, and protein processing in the endoplasmic reticulum were found enriched at 6 hpi (Supplementary Figure 3B), while photosynthesis, cysteine and methionine metabolism, phenylpropanoid biosynthesis, and glutathione metabolism were enriched at 12 hpi (Supplementary Figure 3C). Moreover, the analysis of the DEGs associated with the photosystems I and II revealed that the cascade genes in charge of the photosynthetic system were downregulated after inoculation, reducing the plant's photosynthetic capacity (Supplementary Figure 3D). In addition, the plant hormone signal transduction, glycometabolism, and photosynthetic light reactions were significantly enriched, suggesting the role of the DEGs associated with these pathways in maize's response to *U. maydis* infection. Based on this result, the present study proposed that the light reactions, Calvin cycle, chloroplast, and photorespiration played significant roles in the invaded leaves at 12 hpi, confirming that the biotrophic growth of *U. maydis*–induced carboxylate metabolism *via* photosynthesis (Supplementary Figure 4).

**Figure 7:**
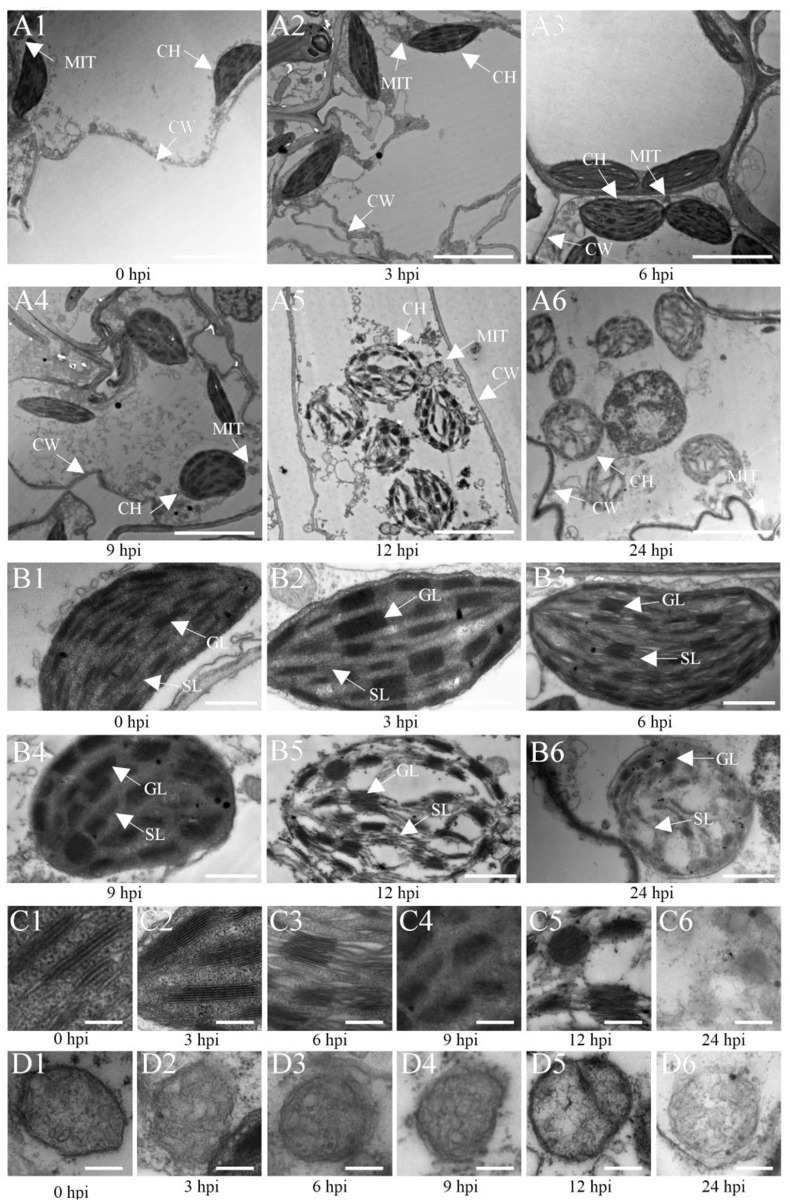
Transmission electron microscopic (TEM) images of Ye478 leaves infected with *Ustilago maydis*. TEM images show sheath cells and mesophyll cells **(A1–A6)**, chloroplasts of sheath cells **(B1–B6)**, grana of chloroplasts **(C1–C6)**, and mitochondria of chloroplasts **(D1–D6)** at 0, 3, 6, 9, 12, and 24 hpi, respectively. CW, cell wall; CH, chloroplast; MIT, mitochondria; GL, grana lamella; SL, stroma lamella. Scale bars: **(A1–A6)** 5 μm; **(B1–B6)** 2 μm; **(C1–C6,D1–D6)** 0.5 μm.

Furthermore, the analysis of the ultrastructure of Ye478 cells inoculated with *U. maydis* (Figure 7) showed oval-shaped cells with a lamellar structure related to the chloroplasts at 3 hpi that were morphologically similar to that of the control (Figures 7A1, A2). The chloroplasts were slightly swollen with well-stacked thylakoids and piled up small starch particles at 6 hpi (Figure 7A3). Compared with the control, the inoculated samples at 9 hpi had partly swollen chloroplasts with loosely arranged thylakoids (Figure 7A4). The chloroplasts were swollen or impaired at 12 dpi as the disease progressed, with a more loosely arranged thylakoid system (Figure 7A5). Further, the invaded cells of Ye478 exhibited a high level of plasmolysis in the mitochondria (Figure 7A6). In addition, the mitochondrial structure was normal, and the epithelium cell demonstrated integrity before 9 hpi, whereas the membrane and inner carinulae were absent in deformed mitochondria after 9 hpi (Figures 7C1–C6). Gradually, chlorosis appeared with the loss of photosynthetic capacity during hyphal infection compared with the mock-infected samples (Figures 7D1–D6), combined with a decrease in the levels of the photosynthetic pigments from 3 to 24 hpi (Supplementary Table 7).

Furthermore, the antioxidants and secondary metabolites after *U. maydis* inoculation were analyzed. The ROS, including various forms of reduced and reactive molecules such as O_2•_^−^, H_2_O_2_, OH_•_, were analyzed. The production of O_2•_^−^, H_2_O_2_, and OH_•_ significantly increased from 3–24 hpi and peaked at 24 hpi; H_2_O_2_ content was seven times higher than that in the after 0 h (Supplementary Figure 5B). PAL, as a catalyst for the first step of phenylpropanoid metabolism, plays a vital role in plant secondary metabolism. Therefore, the PAL activity in the inoculated leaves of Ye478 was investigated. The result showed an increase throughout the process, with maximum activity at 24 hpi, which was ~50% higher than the initial values (Supplementary Figure 5D). The GSSG and GST levels also rapidly increased with *U. maydis* infection, while the GSH level showed an opposite trend from 3 to 24 hpi; GSH/GSSG attained a maximum between 12 and 24 hpi (Supplementary Figures 5E–G, 6). In addition, the ASA level showed a decrease, while the DHA level showed an increase; ASA/DHA decreased with *U. maydis* infection (Supplementary Figures 5H,I; 6).

**Figure 8:**
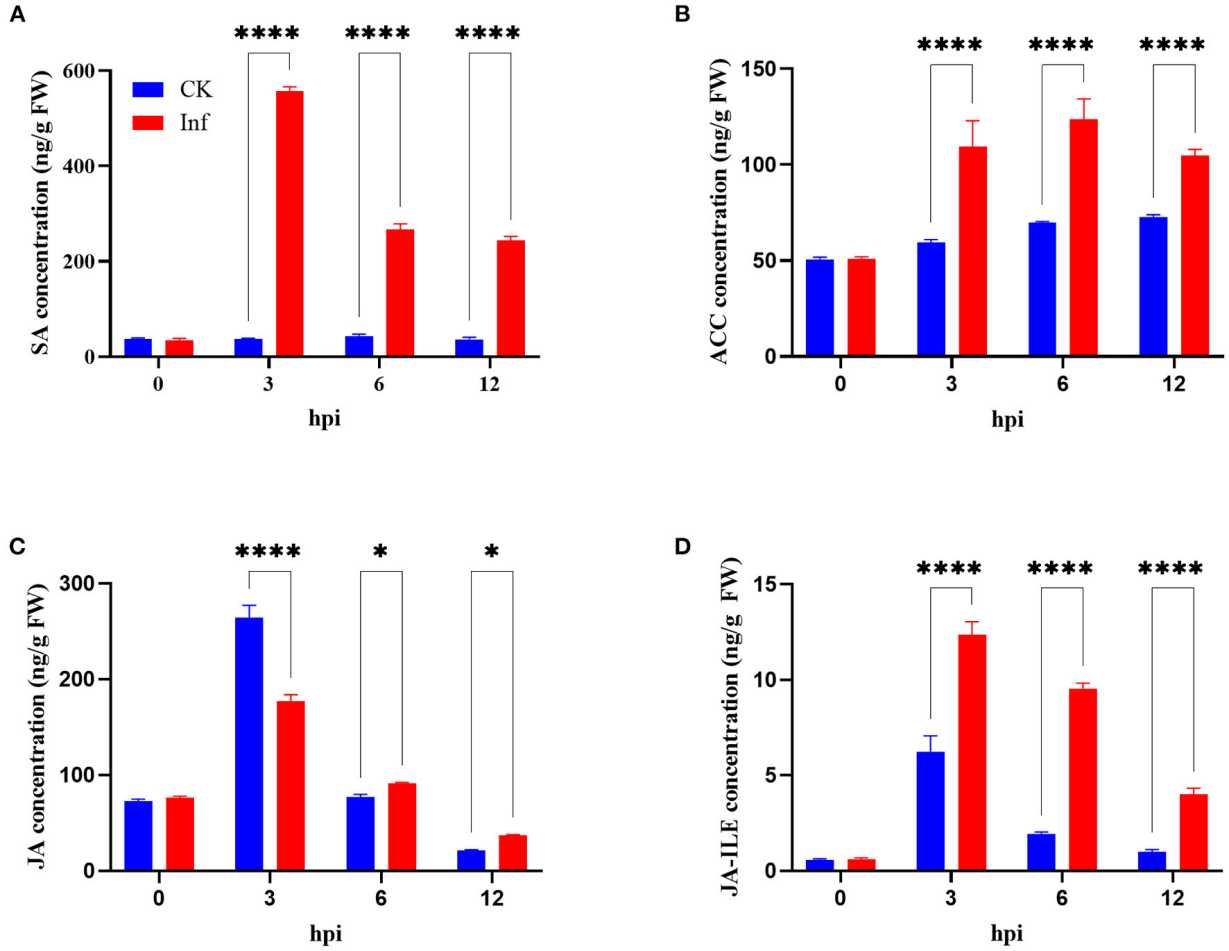
Changes in phytohormone levels in maize after *Ustilago maydis* infection. **(A–D)** Total content of salicylic acid (SA), ethylene precursor (ACC), jasmonate (JA), and jasmonoyl-isoleucine (JA-ILE) in *U. maydis*-infected (red bars) and control leaves (blue bars) at 0, 3, 6, and 12 hpi, respectively. Data represent mean ± SD (three biological replicates from seven leaves). Statistically significant differences between control and *U. maydis*-infected samples determined based on Student's *t-*test are indicated (*P < 0.05; ****P < 0.0001).

This study further analyzed the concentrations of SA, ACC, JA, and JA-ILE in the leaves at 0, 3, 6, and 12 h after *U. maydis* inoculation. Analysis revealed that SA, JA, and JA-ILE were strongly induced, with a peak after 3 h that gradually dropped; all these levels were significantly higher than those in control during the whole infection process (Figures 8A,C,D). Whilst, ACC concentration increased after inoculation and then decreased, the maximum activity appeared at 6 hpi (Figure 8B). After inoculation, the expression levels of genes related to SA, ACC, JA, and JA-ILE biosyntheses also significantly increased (Supplementary Table 4).

### *Gsg* and *ygl-1* mutants increased maize resistance to *U. maydis*

**Figure 9:**
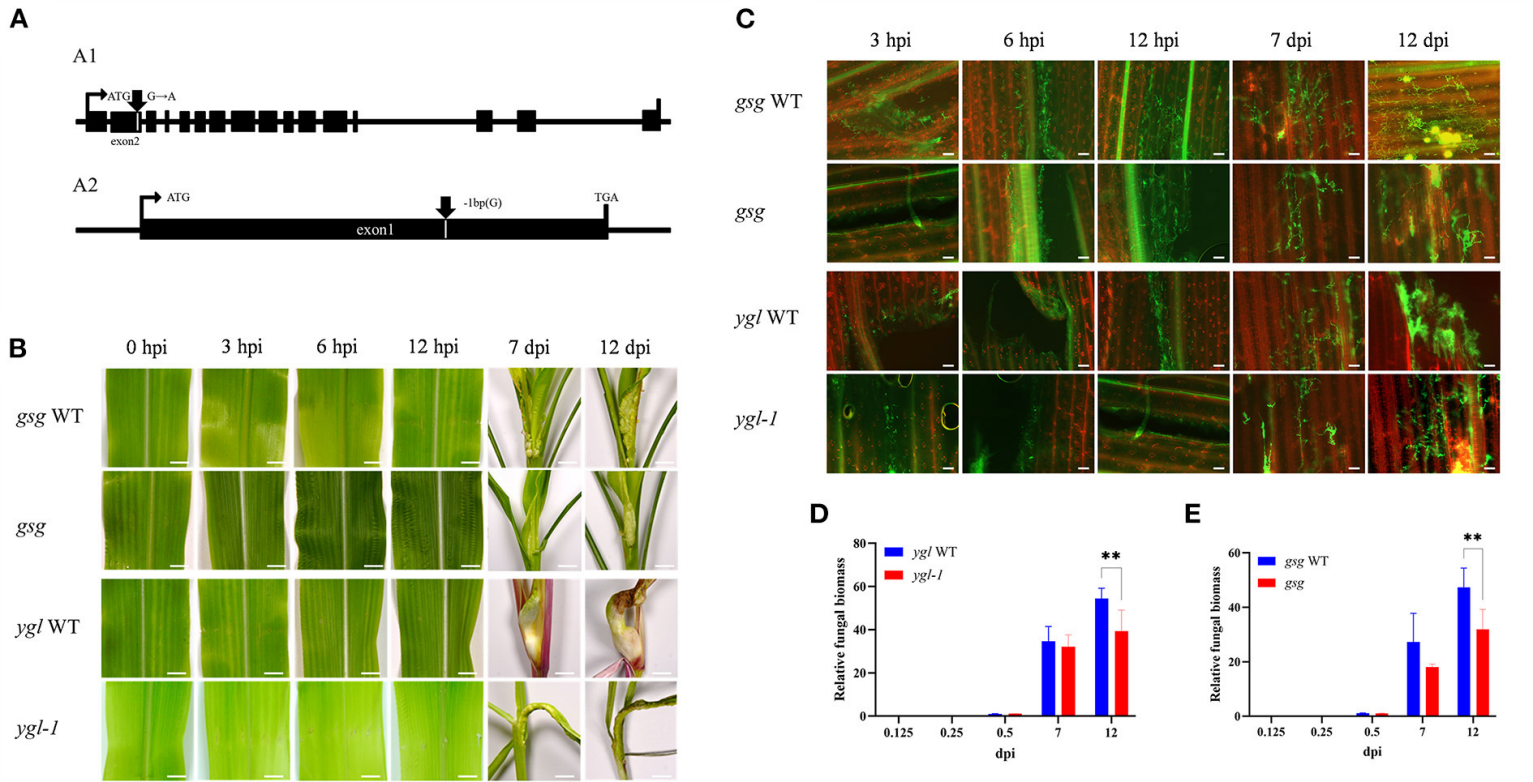
Maize mutants defective in sugar and chlorophyll biosynthesis showed resistance to *Ustilago maydis* infection. **(A)** Schematic diagrams showing the genomic structures of the mutants; **(A1)** transition mutants showing the *gsg* allele with the G/A base transition in the second exons, **(A2)** deletion mutant showing a 1-bp nucleotide deletion in the first exons. **(B)** Comparison of symptoms in the two maize mutants (*gsg* and *ygl-1*) defective in sugar and chlorophyll biosynthesis with the wild-type leaves after *U. maydis* inoculation. Inoculated leaves were collected at 3, 6, 12, 7, and 12 dpi, respectively to compare the phenotypes. Scale bar = 1 cm. **(C)** Growth of *U. maydis* in the mutants and wild-type plants was analyzed by staining with WGA-AF488 (green). The plant cell wall was stained with propidium iodide (red). Scale bar = 50 μm. **(D,E)** Relative fungal biomass of mutants calculated based on the amount of genomic DNA detected by real-time PCR. Plant-specific (GAPDH) and fungal-specific (*ppi*) gene primers were used to analyze WT (blue bars) and mutant (red bars) data at 0.125, 0.25, 0.5, 7, and 12 dpi, respectively. Each error bar represents the SD of three biological replicates. Asterisks indicate statistical significance (*t*-test; **P < 0.01).

The ethyl methane sulfonate (EMS) chemical mutagenesis was carried out in the B73 inbred lines, and the homozygous individuals were screened *via* PCR product sequencing. The B73 pollens were first treated with EMS and self-pollinated to produce M_1_ seeds. Exon capture and Illumina sequencing were used to analyze the genomic DNA of the M_1_ plants. The M_2_ plants were individually inherited from each M_1_ plant, and the M_3_ seeds were procured from a mix of all the M_2_ plants for distribution. The mutations were confirmed following the Genome Analysis Toolkit (GATK) pipeline, and the sequences were uploaded in the public database (http://www.elabcaas.cn/memd/; Lu et al., 2018). The -sucrose galactosyltransferase (*gsg*) mutant was used to evaluate the gene function in the maize defense mechanism against common smut. Bioinformatics analysis showed a G/A base transition in the *gsg* allele in the second exon; the codon change tGg/tAg directly caused premature gene termination (Figure 9A1 and Supplementary Figure 7). On the contrary, yellow-green leaf mutant (*ygl-1*) was isolated from the inbred progenies obtained *via* hybridization of Ye478 and Yuanwu02 (Guan et al., 2016), with a 1-bp nucleotide deletion in the first exon that led to a frameshift mutation, thereby causing premature gene termination (Figure 9A2 and Supplementary Figure 7). Furthermore, *gsg* and *ygl-1* mutants were inoculated with *U. maydis* to investigate the role of hexose and chlorophyll biosynthesis pathways in maize's response to *U. maydis* infection. As expected, these mutants displayed fewer tumors and reduced disease incidence than the WT control 3, 6, and 12 h after *U. maydis* inoculation, and until 7 and 12 dpi (Figure 9B). WGA-AF488/PI co-staining demonstrated reduced fungal growth within the leaf tissues throughout the infection process at 3, 6, and 12 hpi and 7 and 12 dpi. In the WT, fungal aggregates and dotted hyphae proliferated, whereas the size and the number of fungal aggregates increased in the mutant (Figure 9C). Further, the relative fungal biomass was assessed by real-time PCR to delineate fungal intrusion in the WT and mutant plants. At 3, 6, and 12 hpi and 7 and 12 dpi, the relative fungal biomass of mutants was significantly lower than that of the WT (Figures 9D,E). These data indicated that hexose and chlorophyll were essential during *U. maydis* invasion in maize, which could restrict fungal intrusion and improve plant resistance.

## Discussion

### Initial phase of *U. maydis* development occurred 3 h after inoculation

The present study found that *U. maydis* infection occurred for 14 days from host penetration to fungal spore release. The analysis revealed that the hyphae penetrated the plant surface at 24 hpi, reached the bundle sheath cells at 2 dpi, and formed tumors after 7 dpi. Further, the hyphae spread severely and appeared swollen by 7 dpi, the tumor maturation was completed after 12 dpi, and the spore aggregates formed after 14 dpi (Matei and Doehlemann, 2016; Lanver et al., 2018; Schurack, 2021). The researchers found that SG200 cells formed filaments and appressoria at 12 hpi, and the attacked epidermis cell of maize collapsed, with disintegrated membrane structures (Doehlemann et al., 2008a; Skibbe et al., 2010; Ma et al., 2018; Schmitz et al., 2020). Certainly, the hyphal infection started from 1 dpi (Skibbe et al., 2010; Matei and Doehlemann, 2016) or 2 dpi (Gao et al., 2013; Redkar et al., 2015; Matei et al., 2018). However, the analysis of the histiocytic changes in response to infection demonstrated that the mitosis of invading strain cells began from 4 hpi, and the fungal hyphae intruded the host plasma membrane and formed a biotrophic interface (Figure 1C). Probably, *U. maydis* exchanged nutrients, signals, and effectors with the host *via* a tight interaction zone. The SEM results also showed that fungal mitosis occurred at the injection site at 4 hpi (Figure 2C), and the hyphae formed appressoria and penetrated host cells as early as at 6 hpi, indicating a special biotrophic interaction between the pathogen and the plant cell (Figures 2D,E). Thus, the artificial inoculation revealed that the start of *U. maydis* infection occurred at 3 hpi. In addition, the GO analysis of the DEGs indicated that plasma membrane, oxidation-reduction, and JA-signaling pathway-related genes were enriched at 3 hpi in the infected samples compared with the control (Supplementary Tables 2, 3). Similarly, significant differences were detected in the ROS system and phytohormones (Supplementary Figures 5, 7). These observations collectively indicated that 3 hpi was a crucial time point when Ye478 allowed fungal development and showed a defense response.

### Physiological changes during the early response to *U. maydis* infection

*Ustilago maydis* infection leads to extensive physiological and developmental changes in the host's immunity function, stress response, and redox regulation, indicating pathogen recognition and early defense response. ROS evoke defense and programmed cell death at the site of pathogen attack, preventing further spread of the biotrophic pathogen (Apel and Hirt, 2004). The levels of O_2•_^−^, H_2_O_2_, and OH_•_ increased significantly from 3 to 24 hpi, with a peak accumulation at 24 hpi (Supplementary Figures 5A–C). Moreover, the levels of H_2_O_2_ were three times more than that of the control during *U. maydis* infection (Supplementary Figure 5B). Although ROS worked as signal molecules in plants after fungal infection, the oxidative stress generated by ROS was fatal to the host. Consequently, the host evolved to clear up ROS using glutathione, alkaloids, flavonoids, ASA, carotenoids, and antioxidant enzymes (Lehmann et al., 2015). This study found that *U. maydis* inoculation induced the production of O_2•_^−^, H_2_O_2_, and OH_•_, followed by an increase in PAL, GSSG, GST, and DHA levels, with the highest induction at 24 hpi (Supplementary Figures 7D–G). In contrast, the reduced GSH and ASA levels were detected during the process (Supplementary Figures 7H,I). The findings of this study indicated that GST probably participated in scavenging the oxygen radicals, resulting from respiratory processes in the plant cells, consistent with the expression of PR genes (Gayoso et al., 2010). Plant endohormones, including JA, SA, and ET, regulated pathogen attack (Doehlemann et al., 2008a; Rabe et al., 2013). The accumulation of phytohormones led to rapid and specific responses under biotic stress. The present study detected a temporal change in JA and SA levels in response to *U. maydis* infection, with a peak at 3 hpi, which gradually declined. Meanwhile, ACC, the precursor of ET, showed insignificant changes following *U. maydis* infection (Figure 8). Studies also indicated that SA signaling led to ROS production, which were highly toxic and destroyed the invading pathogens (Tamaoki, 2008). Therefore, it was speculated that biotrophic pathogens developed strategies to hold back the accumulation of SA and then reduced ROS levels. Nevertheless, defense is a complicated process wherein the phytohormones mediate direct and indirect responses. However, the biotrophic susceptibility in maize remains unclear. Recently, Darino et al. found that a functional effector of *U. maydis*, jasmonate/ethylene signaling inducer 1 (Jsi1), acted with various members of the plant Topless/Topless related corepressor family and increased the transcription of the ethylene response factor (ERF) branch of the JA/ET signaling pathway, promoting fungal infection (Darino et al., 2021). Previously, Caarls et al. reported that the improvement in SA-signaling was based on the inhibition of JA/ET signaling, which led to increased resistance toward biotrophic interactions. Therefore, biotrophic pathogens have to induce the JA pathway to suppress SA-mediated defense of the host (Caarls et al., 2015). Obviously, pathogens aim to colonize host plants to get nutrients and complete their life cycle. Therefore, they have evolved strategies to manipulate the plant defense-related hormone signaling to increase susceptibility and result in infection. Thus, identifying and functionalizing the genes involved in JA and SA signaling pathways are important to reveal the plant-pathogen interaction process.

### Maize gene expression following *U. maydis* inoculation

Usually, response to biotic stress, including plant pathogens, is primarily regulated at the transcription level (Schurack, 2021). Moreover, resistance is generally considered a quantitative trait regulated by several genes (Schurack et al., 2021). However, the mechanisms underlying the defense against *U. maydis* in maize remain elusive. Studies reported that PS and C4, primary carbon metabolism, protein metabolism and transcription, and RNA-processing contributed to resistance (Doehlemann et al., 2008a). Moreover, cell wall component metabolism, hypothetical protein, monosaccharide metabolism (Matei et al., 2018), glycolysis, amino acids, phenylpropanoid, ROS (Ruan et al., 2021), cell division, and photosynthesis (Schurack et al., 2021) were identified during tumor formation, consistent with our DEGs dataset. In this study, the common smut -susceptible inbred line Ye478 infected with *U. maydis* elicited prominent changes in the expression of defense-related genes, with more upregulated genes than downregulated (Figure 3), generally involved in hormone signal transduction, glycolysis and glycogenesis, and photosynthesis (Supplementary Figure 2). Meanwhile, the *gsg* mutants showed strong resistance than the WT. The relative fungal biomass was also lower in the mutants, indicating the mutants as good models for the isolation of defense-related genes. The mutant *gsg*, an activated sugar donor that catalyzes the formation of a glucosidic bond, is regulated by various abiotic and biotic stresses. This protein was upregulated in tropical and temperate maize lines Suwan-2 and Cim-3 under waterlogging conditions (Yao, 2021). In *U. maydis*, the hyphal growth requires nutrients, which determines biotrophic development and involves organ-specific gene expression of sugar transporters. Seven sucrose transporters (*SUTs*), 23 sugar transport proteins (*STPs*), and 24 sugars will eventually be exported transporters (*SWEETs*) have been identified in the maize genome (Sosso et al., 2019). Among them, 13 genes, including three *ZmSUTs*, seven *ZmSTPs*, and three *ZmSWEETs*, expressed differentially and tissue-specifically during *U. maydis* infection (Skibbe et al., 2010). However, the hexose within the tumor cells acted as an easily accessible carbon source for the fungus and helped expand tumor cells (Horst et al., 2008, 2010a). Moreover, the findings of this study showed that photosynthesis had not been hindered during *U. maydis* induced host responses induced host responses. In the infected tissue, the photosynthesis-related genes were downregulated (Figure 6), and chloroplast and photosynthetic pigments were impaired (Figures 7, 8), accompanied by an increased photosynthetic dysfunction. In addition, the light reactions, Calvin cycle, and photorespiration were strongly induced at the transcriptional level in the infected leaves, suggesting that the biotrophic growth in maize might strongly induce photosynthesis (Supplementary Figures 3, 4). The accelerated metabolic flux for carboxylate production *via* the TCA cycle could either provide sufficient NADPH and ATP to the host cells or increase the respiratory flux due to disabled photo-autotrophy during biotrophic settlement. However, a systematic study analyzing the dynamics in nitrogen metabolism during the interaction of maize with *U. maydis* demonstrated that the TCA cycle worked as a potential provider of nitrogen assimilation with carbon skeletons (Horst et al., 2010a,b). The *ygl-1* mutant with a defect in chlorophyll biosynthesis displayed high susceptibility to *Rhizoctonia solani* Kühn, indicating the vital role of chlorophyll in resisting *R. solani* infection in maize (Cao et al., 2022). However, for *U. maydis* infection, the contrasting phenotypes of the *ygl-1* mutant improved resistance, and an opposite phenotype was detected during *R. solani* infection, possibly indicating different PR mechanisms. Meanwhile, both pathogens induced specific changes in primary metabolism and reduced chloroplast and photosynthetic functions. The recruitment of photosynthesis in the mutant during tumor formation might result in the changes in carbon allocation that limited the nutrient supply, thereby cutting down food supply and reducing the proliferation of the fungi. Therefore, identifying the differences in nutrient competition between the mutants and WT during *U. maydis* infection is important. In general, differential and redirected nutrient supply played a crucial role in establishing an interaction between maize and *U. maydis*. The results of this study provided a foundation for further studies on the defense mechanisms against *U. maydis* so as to mine durable resistance genes.

## Conclusion

The present study characterized early infection response in the leading maize inbred line Ye478 infected by *U. maydis*. The histological and cytological observations demonstrated that *U. maydis* began to grow gradually into the host cells from the wound site after 6 h of inoculation. The analysis of the transcriptome changes at 0, 3, 6, and 12 hpi revealed that diverse biological pathways, including hormone signaling, glycometabolism, and photosynthesis, were altered after infection. Among these pathways, JA, SA, ET, or ABA signaling pathways, glycolysis/gluconeogenesis, and photosystems I and II were closely correlated with defense response. Further studies using the WT plants and *gsg* and *ygl-1* mutants showed that these two *U. maydis* infection-induced genes negatively regulated maize defense against common smut. In conclusion, the findings of this study provided valuable information about maize defense against *U. maydis* and suggested that chlorophyll biosynthesis and sugar transport were important for resistance.

## Data availability statement

The original contributions presented in the study are included in the article/Supplementary material, further inquiries can be directed to the corresponding author.

## Author contributions

WD and YX discussed and designed the experiment. KZ, YL, WZ, and YJ performed the experiment. YW and YM analyzed the data. WD, KZ, and XL drafted the manuscript. All authors have read and approved the manuscript.

## Funding

Much appreciated financial support was provided by the National Natural Science Foundation of China (No. 32272152) and Basic Research Project of Education Department of Liaoning Province (No. LSNJC202017), Introduced Talents Funding Project of Shenyang Agricultural University (No. 20153005), and Shenyang science and technology planning project (No. 21110316).

## Conflict of interest

The authors declare that the research was conducted in the absence of any commercial or financial relationships that could be construed as a potential conflict of interest.

## Publisher's note

All claims expressed in this article are solely those of the authors and do not necessarily represent those of their affiliated organizations, or those of the publisher, the editors and the reviewers. Any product that may be evaluated in this article, or claim that may be made by its manufacturer, is not guaranteed or endorsed by the publisher.
