# Supplementary material for: Early infection response of fungal biotroph Ustilago maydis in maize
Source: Front Plant Sci. 2022 Sep 9;13:970897. doi: 10.3389/fpls.2022.970897 (PMC9504671; doi:10.3389/fpls.2022.970897)
Supplement: Supplementary file 1 [file Data_Sheet_1.docx]

## Supplementary Figures

**
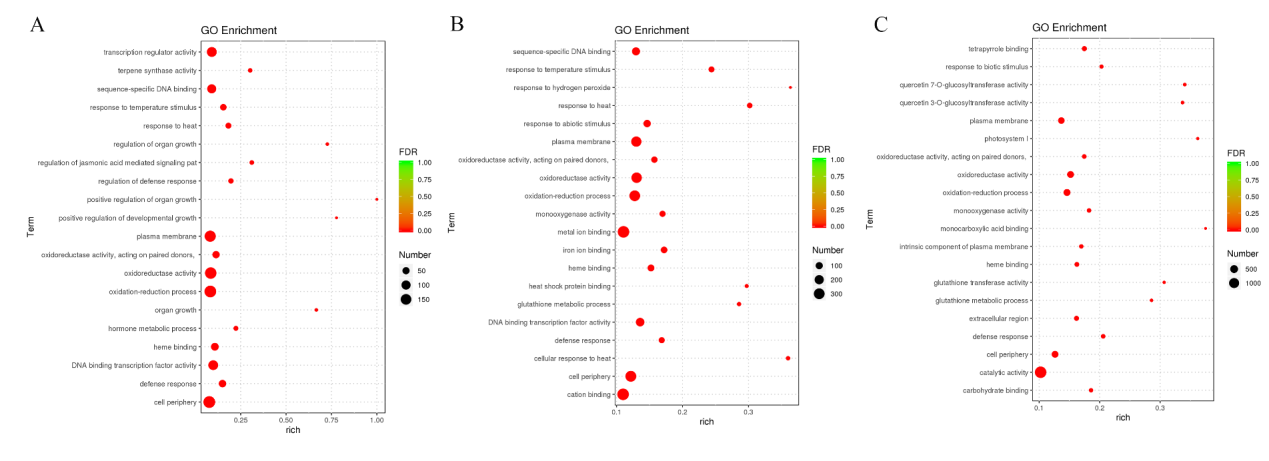
 Supplemental Figure. 1** GO analysis of differentially expressed genes (DEGs). (A–C) GO analysis of DEGs identified at 3 h, 6 h and 12 h after *U. maydis* (Inf) infection vs. control (injected with distilled water, CK). Rich represents the ratio of the number of DEGs annotated with a GO term relative to the total number of genes annotated with this term. FDR represents the false discovery rate.

**
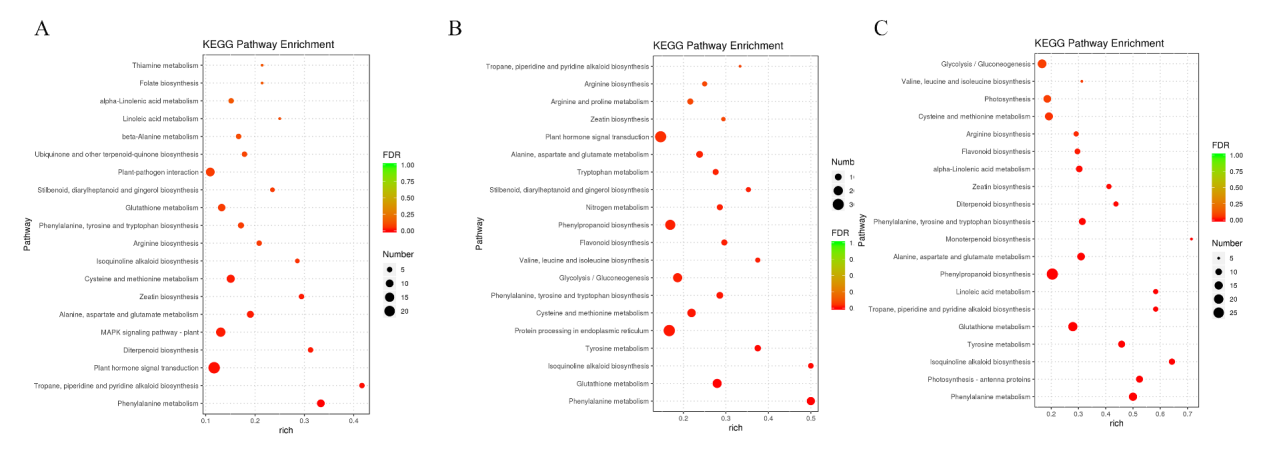
 Supplemental Figure. 2** KEGG analysis of differentially expressed genes (DEGs) of CK vs. Inf samples at 3 h, 6 h, and 12 h after *Ustilago maydis* inoculation. (A–C) Pathway enrichment analysis of DEGs at 3 h, 6 h, and 12 h after *U. maydis* infection (Inf) vs. CK. The size of the dots represents the ratio of the number of DEGs annotated with a pathway relative to the total number of genes annotated. FDR represents the false discovery rate.

**
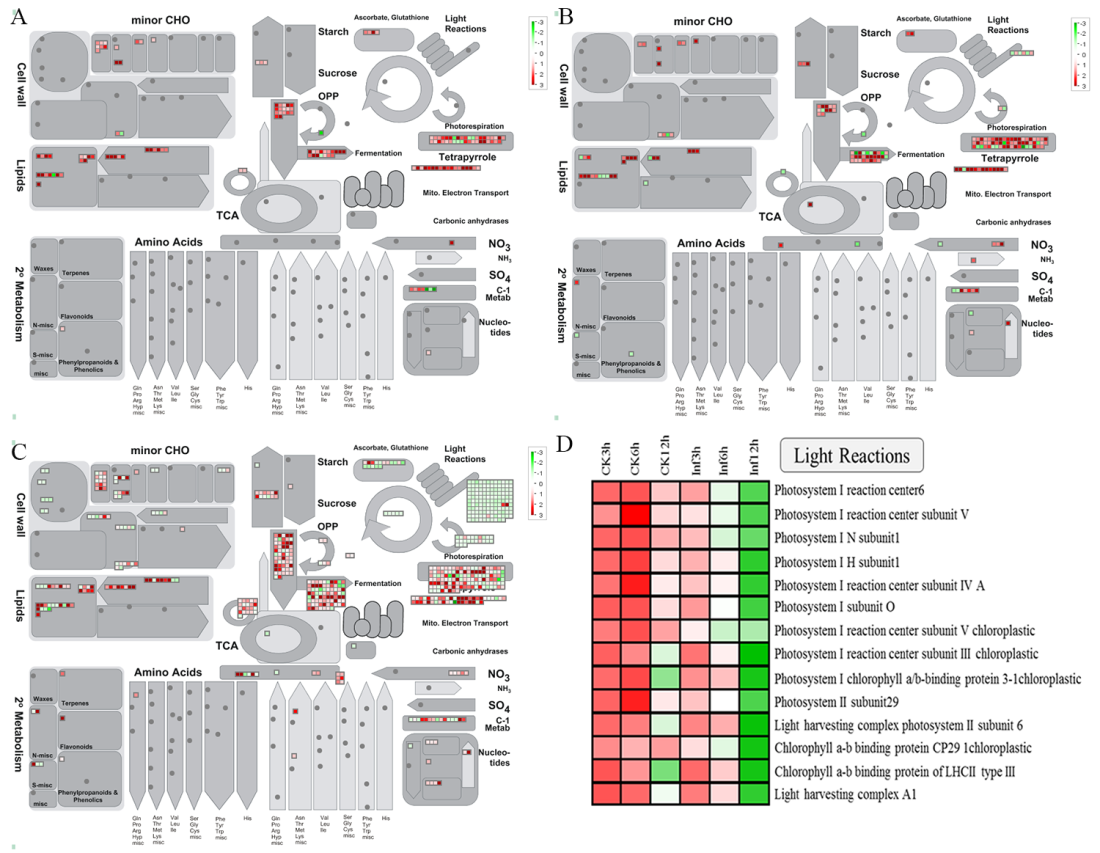
 Supplemental Figure. 3** Mapman overview of the differentially expressed transcripts identified between *Ustilago maydis* infected and masked leaves. The images were obtained using the MapMan software. Various functional categories with differential expression levels were described (less than 0.05 Q value and greater than 2-fold change); green shows downregulation, and red shows upregulation. (A) The primary metabolism changes associated with the differentially expressed genes (DEGs) between the inoculated samples at 3 hpi and control, (B) 6 hpi and control, and (C) 12 hpi and control. (D) DEGs associated with light reactions in the leaf tissues at 3, 6 and 12 hpi compared with the control.

**
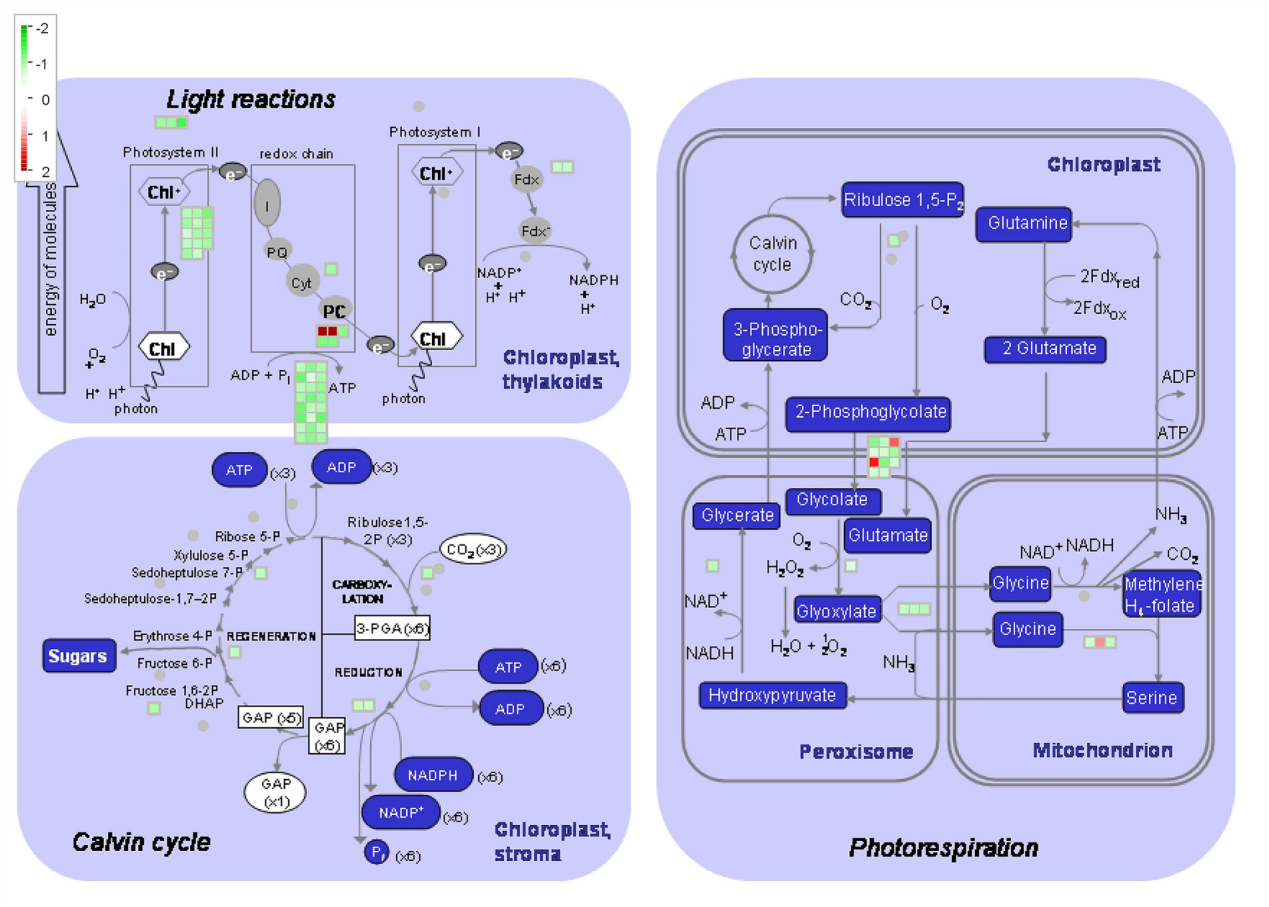
 Supplemental Figure. 4** Overview of photosynthetic changes in the control (CK) and *Ustilago maydis*-inoculated samples (Inf) based on MapMan analysis. Each square and color indicate the fold change based on RNA-seq data. Green square indicates a decrease, and red square indicates an increase in photosynthesis (Light reactions), Calvin cycle, and photorespiration.


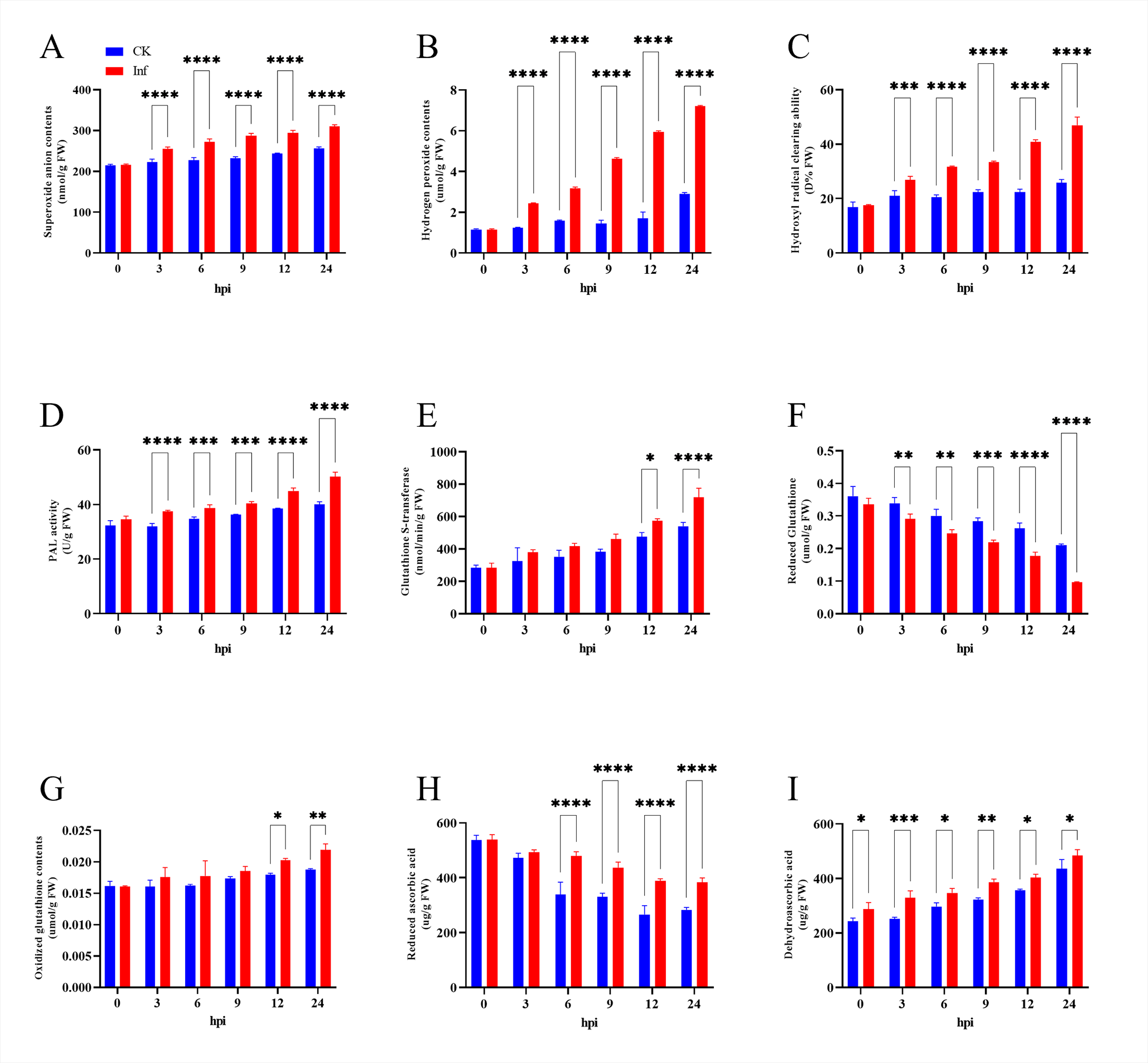


**Supplemental Figure. 5** Changes in the reactive oxygen species (ROS) and secondary metabolites in leaf tissues infected with *Ustilago maydis*. Specimens were collected at 0, 3, 6, 9, 12 and 24 hpi. (A–C) Content of superoxide anion (O_2•_ ^̶^), hydrogen peroxide (H_2_O_2_), and hydroxyl radical (OH_•_). (D–I) Activity of phenylalanine ammonium-lyase (PAL), glutathione S-transferase (GST), including reduced glutathione (GSH) and oxidized glutathione (GSSG), reduced ascorbic acid (ASA), and dehydroascorbic acid (DHA). Blue bars show control leaves, and red bars show *U. maydis*-infected leaves. Error bars indicate the standard deviation of three biological replicates (from seven leaves). Asterisks show statistically significantly differences (*t*-test; *P < 0.05; **P < 0.01; ***P < 0.001; ****P < 0.0001).

**
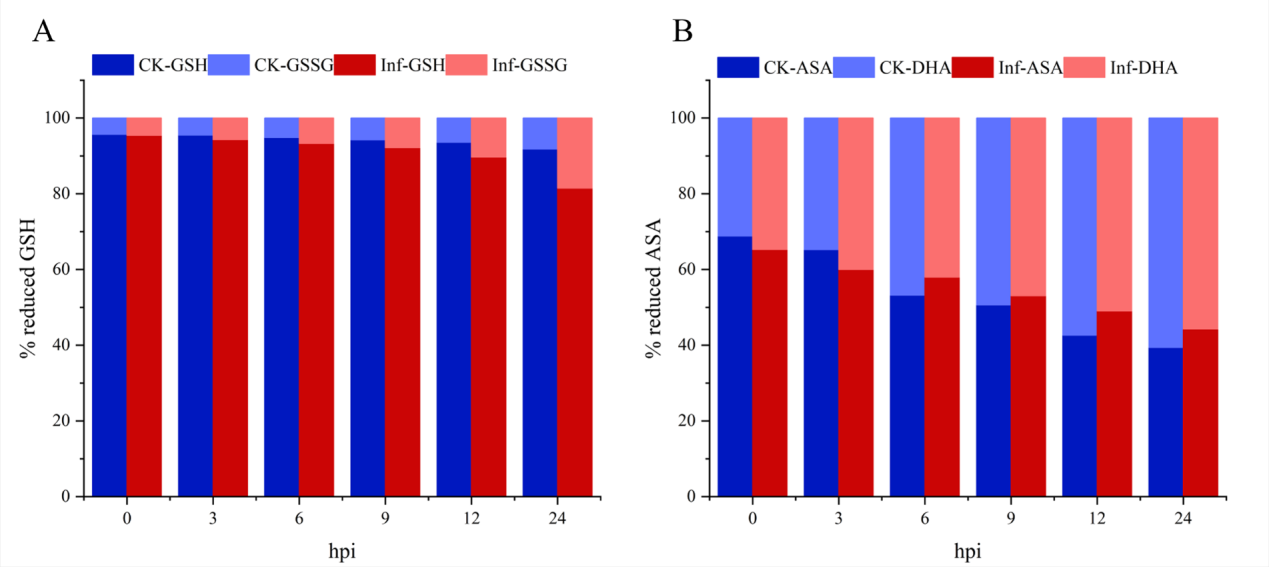
 Supplemental Figure. 6** Antioxidants in response to *Ustilago maydis* infection. (A) Reduced state of the glutathione pool. (B) Reduced state of the ascorbic acid pool. For all graphs, total reduced GSH and ASA state in control leaves (dark blue bars) and *U. maydis*-infected leaves (dark red bars), and total oxidative GSSG and DHA state in control leaves (blue bars) and *U. maydis*-infected levels (pink bars) are shown. Data represent mean values (three biological replicates from seven leaves).

**
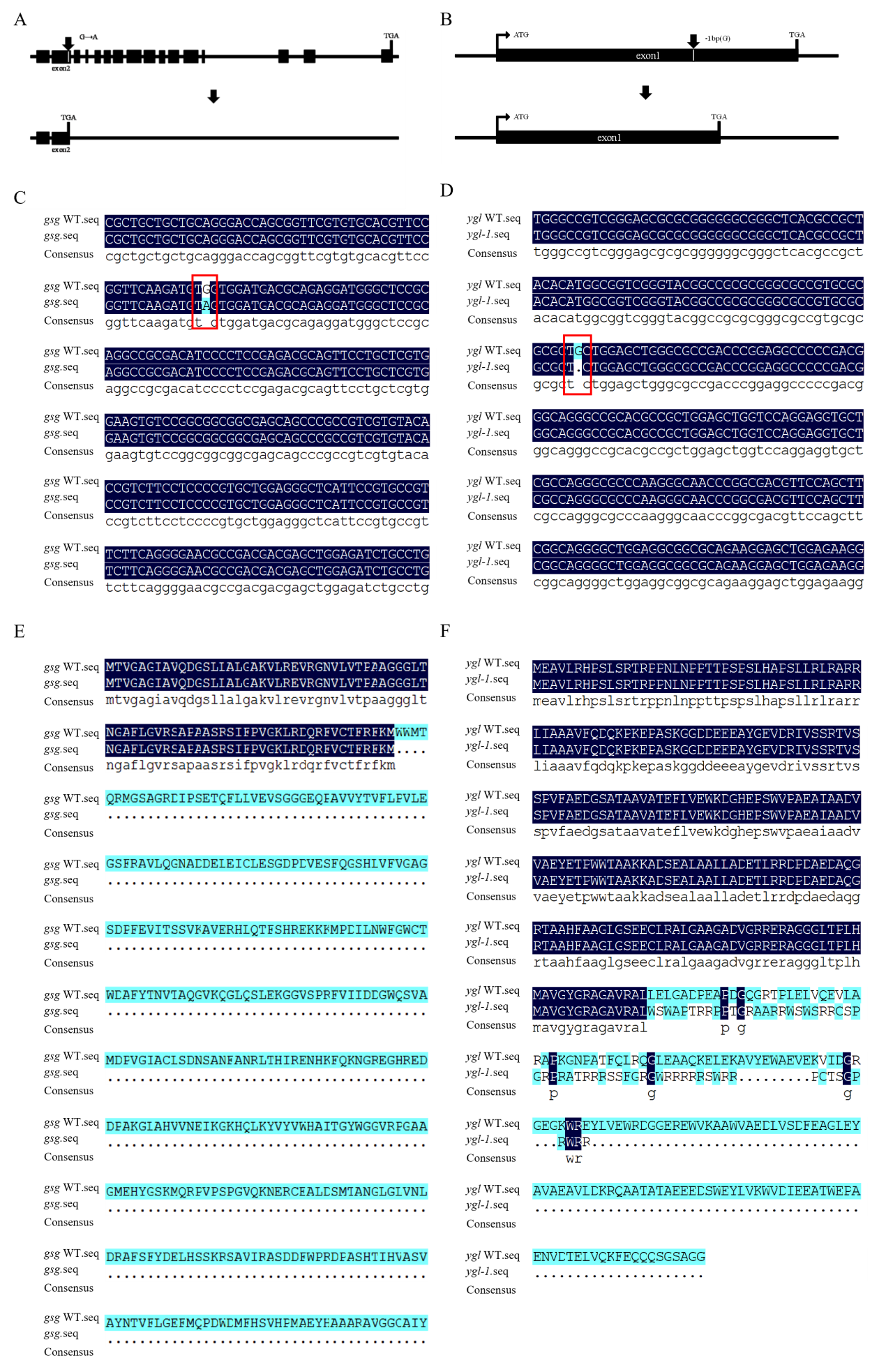
** **Supplemental Figure. 7** Sequence analysis of *gsg* and *ygl-1* mutants. Schematic diagrams show the genomic structures of wild type and mutants: (A) G/A base transition mutant *gsg* (second exon) and (B) base deletion (G) mutant *ygl-1* (first exon). Nucleotide sequence of *gsg* (C) and *ygl-1* (D). The protein sequences of *gsg* (E) and *ygl-1* (F) are shown.

# Supplementary Tables

**Supplementary Table 1** Primers used for qRT-PCR assay

| **Gene ID** | **F** | **R** | **Gene description** |
| --- | --- | --- | --- |
| Zm00001d016924 | CGCGAGCTGATAAGTAGCAG | GTAGTGACGCTTCAGGAGTTC | Ethylene insensitive 3-like 5 protein |
| Zm00001d011377 | GGTTGGCAAAATCTACGAAGTT | TGCAGATCTTTCTCTGTGTTCT | Jasmonic acid-amido synthetase JAR1 |
| Zm00001d031303 | GTAGGAGGCTTTGTGTGTTTTC | CATCCTCACCACTGAATCTCA | probable galactinol-sucrose galactosyl transferase1 |
| Zm00001d028230 | GATGTCAAAGGGCATGCG | TTTGCACTCTCCAGCCATAATC | sugar transport protein13 |
| Zm00001d042337 | TATGCATCCACTGATTGGTAGT | TAAACTTACGCACACACCTACC | Nucleotide-diphospho-sugar transferase family protein |
| Zm00001d023941 | ACCGTGTAGCTACAGGTATAGA | TGCTTTATAGATGGCAACGGTA | Carbohydrate transporter/sugar porter |
| Zm00001d042453 | TTTCCAGCATCAATGTCGAGAA | CCTTGAGGACATCAACAAAAGG | Aldolase1 |
| Zm00001d043607 | ATGAAATGGTAGGAAAGGACGT | CATTAGTGCCAGTACCCAGTAT | Hexokinase6 |
| Zm00001d012103 | GGTATCGGCAAAAGCCATTTAT | TAAATCGGTATGTTCGGAACCA | Aldolase2 |
| Zm00001d038775 | GTCTTTTGGTTTTGATACGGCT | CGACTAGCAAGAGTAGCATACA | ATP-dependent 6-phosphofructokinase3 |
| Zm00001d003767 | CCATTATTCGTTGCAGTAGTCG | GAAACGTCATGCAGGTGATC | Photosystem I subunit O |
| Zm00001d018797 | CCAATGGACATATAGCTCCTGT | GTTTTAACCGCTTGCTAATTGC | Photosystem I reaction center6 |
| Zm00001d041819 | CTTGTCGACCCTAGATATGTCC | AGCCTCCAATGTCTTAAACTGA | Photosystem I N subunit1 |
| Zm00001d038984 | CTACTTTGACCTCGACGACATC | TCTCGAAGAACTTGCTCTGTAG | Photosystem I H subunit1 |
| Zm00001d050403 | TTCGTGTGATGCGTTCTTAATC | CGTTGAGGTTTTAGAAGTCGTG | Chlorophyll a-b binding protein4 |
| Zm00001d021784 | GATCTGTACCATGAACGAATGC | ACATGGAATGGAAGAGCAAAAC | Chlorophyll a-b binding protein of LHCII typeIII |
| Zm00001d031303 | GTAGGAGGCTTTGTGTGTTTTC | CATCCTCACCACTGAATCTCA | probable galactinol-sucrose galactosyl transferase1 |
| ppi | ACATCGTCAAGGCTATCG | AAAGAACACCGGACTTGG | *U. maydis* specific primer |
| GAPDH | CTTCGGCATTGTTGAGGGTTTG | TCCTTGGCTGAGGGTCCGTC | Plant specific primer |

**Supplementary Table 2** Analysis of differentially expressed genes at 3 h, 6 h, and 12 h after *U. maydis* inoculation

| **Control** | **Treat** | **Up-regulated** | **Down-regulated** | **Total** |
| --- | --- | --- | --- | --- |
| CK3h | Inf3h | 1449 | 394 | 1843 |
| CK6h | Inf6h | 1981 | 1093 | 3074 |
| CK12h | Inf12h | 2184 | 895 | 3079 |
| CK3h | CK6h | 363 | 208 | 571 |
| CK6h | CK12h | 996 | 966 | 1962 |
| CK3h | CK12h | 757 | 588 | 1345 |
| Inf3h | Inf6h | 1067 | 657 | 1724 |
| Inf6h | Inf12h | 1087 | 766 | 1853 |
| Inf3h | Inf12h | 1700 | 1286 | 2986 |

**Supplementary Table 3** GO terms significantly enriched by the differentially expressed genes (DEGs) between CK and Inf samples at 3 h, 6 h, and 12 h after *Ustilago maydis* inoculation

| **Item** | **GO.ID** | **Term** | **Up-number** | **Down-number** | **DEG-number** | **P-value** |
| --- | --- | --- | --- | --- | --- | --- |
| CK3h vs. Inf3h | GO:0005886 | plasma membrane | 138 | 30 | 168 | 7.6E-13 |
|  | GO:0071944 | cell periphery | 159 | 36 | 195 | 2.8E-12 |
|  | GO:0006952 | defense response | 52 | 4 | 56 | 7E-12 |
|  | GO:0003700 | DNA binding transcription factor activity | 99 | 21 | 120 | 5.1E-11 |
|  | GO:0016491 | oxidoreductase activity | 156 | 25 | 181 | 2E-10 |
|  | GO:0046622 | positive regulation of organ growth | 7 | 0 | 7 | 1.5E-09 |
|  | GO:0055114 | oxidation-reduction process | 165 | 27 | 192 | 2.2E-09 |
|  | GO:0140110 | transcription regulator activity | 102 | 21 | 123 | 4.9E-09 |
|  | GO:0009266 | response to temperature stimulus | 31 | 7 | 38 | 0.000000007 |
|  | GO:0009408 | response to heat | 24 | 5 | 29 | 9.8E-09 |
|  | GO:0046620 | regulation of organ growth | 7 | 1 | 8 | 0.000000011 |
|  | GO:0035265 | organ growth | 7 | 1 | 8 | 0.000000032 |
|  | GO:0048639 | positive regulation of developmental growth | 7 | 0 | 7 | 0.000000047 |
|  | GO:2000022 | regulation of jasmonic acid mediated signaling pathway | 14 | 0 | 14 | 0.000000068 |
|  | GO:0020037 | heme binding | 58 | 5 | 63 | 0.00000011 |
|  | GO:0016705 | oxidoreductase activity, acting on paired donors, with incorporation or reduction of molecular oxygen | 46 | 7 | 53 | 0.00000017 |
|  | GO:0010333 | terpene synthase activity | 12 | 1 | 13 | 0.00000022 |
| CK6h vs. Inf6h | GO:0009408 | response to heat | 45 | 3 | 48 | 1.6E-14 |
|  | GO:0005886 | plasma membrane | 192 | 74 | 266 | 2.7E-14 |
|  | GO:0009266 | response to temperature stimulus | 52 | 8 | 60 | 3.8E-13 |
|  | GO:0071944 | cell periphery | 218 | 90 | 308 | 9.1E-13 |
|  | GO:0016491 | oxidoreductase activity | 199 | 79 | 278 | 4.2E-12 |
|  | GO:0055114 | oxidation-reduction process | 211 | 85 | 296 | 4.3E-11 |
|  | GO:0034605 | cellular response to heat | 26 | 1 | 27 | 1E-10 |
|  | GO:0005506 | iron ion binding | 51 | 29 | 80 | 4.2E-09 |
|  | GO:0003700 | DNA binding transcription factor activity | 108 | 57 | 165 | 9.3E-09 |
|  | GO:0006749 | glutathione metabolic process | 21 | 5 | 26 | 0.000000057 |
|  | GO:0004497 | monooxygenase activity | 41 | 25 | 66 | 0.00000016 |
|  | GO:0009628 | response to abiotic stimulus | 79 | 30 | 109 | 0.00000016 |
|  | GO:0006952 | defense response | 54 | 9 | 63 | 0.00000064 |
|  | GO:0043565 | sequence-specific DNA binding | 87 | 52 | 139 | 0.0000021 |
|  | GO:0006457 | protein folding | 48 | 7 | 55 | 0.0000078 |
|  | GO:0010817 | regulation of hormone levels | 12 | 12 | 24 | 0.0001 |
|  | GO:2000022 | regulation of jasmonic acid mediated signaling pathway | 10 | 0 | 10 | 0.00547 |
| CK12h vs. Inf12h | GO:0016491 | oxido reductase activity | 246 | 78 | 324 | 4.1E-23 |
|  | GO:0055114 | oxidation-reduction process | 257 | 82 | 339 | 1.4E-20 |
|  | GO:0003824 | catalytic activity | 1113 | 339 | 1452 | 4.7E-18 |
|  | GO:0005886 | plasma membrane | 200 | 80 | 280 | 1.2E-17 |
|  | GO:0071944 | cell periphery | 228 | 92 | 320 | 3.7E-15 |
|  | GO:0046906 | tetrapyrrole binding | 81 | 30 | 111 | 3.7E-12 |
|  | GO:0006952 | defense response | 66 | 11 | 77 | 4.7E-12 |
|  | GO:0005576 | extracellular region | 74 | 36 | 110 | 1.2E-11 |
|  | GO:0080044 | quercetin 7-O-glucosyltransferase activity | 27 | 4 | 31 | 2.2E-11 |
|  | GO:0080043 | quercetin 3-O-glucosyltransferase activity | 27 | 4 | 31 | 3.1E-11 |
|  | GO:0016705 | oxidoreductase activity, acting on paired donors, with incorporation or reduction of molecular oxygen | 67 | 14 | 81 | 3.3E-09 |
|  | GO:0009522 | photosystem I | 1 | 20 | 21 | 3.5E-09 |
|  | GO:0004497 | monooxygenase activity | 57 | 14 | 71 | 4.5E-09 |
|  | GO:0020037 | heme binding | 80 | 15 | 95 | 6.9E-09 |
|  | GO:0030246 | carbohydrate binding | 56 | 7 | 63 | 0.000000016 |
|  | GO:0031226 | intrinsic component of plasma membrane | 44 | 21 | 65 | 0.000000032 |
|  | GO:0033293 | monocarboxylic acid binding | 13 | 5 | 18 | 0.000000063 |
|  | GO:0006749 | glutathione metabolic process | 23 | 3 | 26 | 0.000000075 |
|  | GO:0004364 | glutathione transferase activity | 21 | 2 | 23 | 0.000000078 |
|  | GO:0051346 | negative regulation of hydrolase activity | 29 | 2 | 31 | 0.000000087 |
|  | GO:0098542 | defense response to other organism | 36 | 7 | 43 | 0.000000088 |
|  | GO:0051213 | dioxygenase activity | 38 | 5 | 43 | 0.000000094 |
|  | GO:0016758 | transferase activity, transferring hexosyl groups | 78 | 17 | 95 | 0.00000024 |
|  | GO:0009521 | photosystem | 1 | 27 | 28 | 0.00000029 |
|  | GO:0009768 | photosynthesis, light harvesting in photosystem I | 1 | 12 | 13 | 0.0000011 |
|  | GO:0016168 | chlorophyll binding | 1 | 15 | 16 | 0.0000021 |
|  | GO:0009765 | photosynthesis, light harvesting | 1 | 13 | 14 | 0.000015 |
|  | GO:0009570 | chloroplast stroma | 18 | 38 | 56 | 0.000019 |
|  | GO:0009523 | photosystem II | 1 | 19 | 20 | 0.000044 |
|  | GO:0009628 | response to abiotic stimulus | 60 | 34 | 94 | 0.00057 |

**Supplementary Table 4** KEGG pathways enriched by differentially expressed genes (DEGs) of CK vs. Inf samples at 3 h, 6 h, and 12 h after *Ustilago maydis* inoculation.

|  |  | Pathway | Pathway ID | Up number | Down number | DEG number | Total number | P value |
| --- | --- | --- | --- | --- | --- | --- | --- | --- |
| CK3h vs. Inf3h |  | Phenylalanine metabolism | zma00360 | 10 | 0 | 10 | 30 | 2.14E-06 |
|  |  | Tropane, piperidine and pyridine alkaloid biosynthesis | zma00960 | 5 | 0 | 5 | 12 | 0.0003 |
|  |  | Plant hormone signal transduction | zma04075 | 22 | 2 | 24 | 205 | 0.0003 |
|  |  | Diterpenoid biosynthesis | zma00904 | 4 | 1 | 5 | 16 | 0.0012 |
|  |  | MAPK signaling pathway - plant | zma04016 | 13 | 2 | 15 | 115 | 0.0012 |
|  |  | Alanine, aspartate and glutamate metabolism | zma00250 | 8 | 0 | 8 | 42 | 0.0016 |
|  |  | Zeatin biosynthesis | zma00908 | 4 | 1 | 5 | 17 | 0.0016 |
|  |  | Cysteine and methionine metabolism | zma00270 | 10 | 1 | 11 | 73 | 0.0017 |
|  |  | Isoquinoline alkaloid biosynthesis | zma00950 | 4 | 0 | 4 | 14 | 0.0055 |
|  |  | Arginine biosynthesis | zma00220 | 5 | 0 | 5 | 24 | 0.0081 |
|  |  | Phenylalanine, tyrosine and tryptophan biosynthesis | zma00400 | 6 | 0 | 6 | 35 | 0.0102 |
|  |  | Glutathione metabolism | zma00480 | 8 | 1 | 9 | 68 | 0.0104 |
|  |  | Stilbenoid, diarylheptanoid and gingerol biosynthesis | zma00945 | 3 | 1 | 4 | 17 | 0.0114 |
|  |  | Plant-pathogen interaction | zma04626 | 13 | 0 | 13 | 119 | 0.0116 |
|  |  | Ubiquinone and other terpenoid-quinone biosynthesis | zma00130 | 5 | 0 | 5 | 28 | 0.0158 |
|  |  | beta-Alanine metabolism | zma00410 | 5 | 0 | 5 | 30 | 0.0209 |
|  |  | Linoleic acid metabolism | zma00591 | 3 | 0 | 3 | 12 | 0.0239 |
|  |  | alpha-Linolenic acid metabolism | zma00592 | 5 | 0 | 5 | 33 | 0.0306 |
|  |  | Folate biosynthesis | zma00790 | 1 | 2 | 3 | 14 | 0.0366 |
|  |  | Thiamine metabolism | zma00730 | 1 | 2 | 3 | 14 | 0.0366 |
|  |  | Tyrosine metabolism | zma00350 | 4 | 0 | 4 | 24 | 0.0378 |
|  |  | Phenylpropanoid biosynthesis | zma00940 | 13 | 0 | 13 | 142 | 0.0429 |
| CK6h vs. Inf6h |  | Phenylalanine metabolism | zma00360 | 14 | 1 | 15 | 30 | 9.98E-09 |
|  |  | Glutathione metabolism | zma00480 | 17 | 2 | 19 | 68 | 6.85E-06 |
|  |  | Isoquinoline alkaloid biosynthesis | zma00950 | 7 | 0 | 7 | 14 | 0.0001 |
|  |  | Tyrosine metabolism | zma00350 | 7 | 2 | 9 | 24 | 0.0002 |
|  |  | Protein processing in endoplasmic reticulum | zma04141 | 27 | 4 | 31 | 186 | 0.0007 |
|  |  | Cysteine and methionine metabolism | zma00270 | 15 | 1 | 16 | 73 | 0.0008 |
|  |  | Phenylalanine, tyrosine and tryptophan biosynthesis | zma00400 | 10 | 0 | 10 | 35 | 0.0009 |
|  |  | Glycolysis / Gluconeogenesis | zma00010 | 18 | 1 | 19 | 102 | 0.0021 |
|  |  | Valine, leucine and isoleucine biosynthesis | zma00290 | 6 | 0 | 6 | 16 | 0.0021 |
|  |  | Flavonoid biosynthesis | zma00941 | 6 | 2 | 8 | 27 | 0.0022 |
|  |  | Phenylpropanoid biosynthesis | zma00940 | 18 | 6 | 24 | 142 | 0.0023 |
|  |  | Nitrogen metabolism | zma00910 | 6 | 2 | 8 | 28 | 0.0029 |
|  |  | Stilbenoid, diarylheptanoid and gingerol biosynthesis | zma00945 | 4 | 2 | 6 | 17 | 0.0030 |
|  |  | Tryptophan metabolism | zma00380 | 6 | 2 | 8 | 29 | 0.0036 |
|  |  | Alanine, aspartate and glutamate metabolism | zma00250 | 9 | 1 | 10 | 42 | 0.0039 |
|  |  | Plant hormone signal transduction | zma04075 | 20 | 10 | 30 | 205 | 0.0067 |
|  |  | Zeatin biosynthesis | zma00908 | 5 | 0 | 5 | 17 | 0.0157 |
|  |  | Arginine and proline metabolism | zma00330 | 7 | 1 | 8 | 37 | 0.0171 |
|  |  | Arginine biosynthesis | zma00220 | 6 | 0 | 6 | 24 | 0.0188 |
|  |  | Tropane, piperidine and pyridine alkaloid biosynthesis | zma00960 | 4 | 0 | 4 | 12 | 0.0192 |
|  |  | C5-Branched dibasic acid metabolism | zma00660 | 3 | 0 | 3 | 7 | 0.0204 |
|  |  | Monoterpenoid biosynthesis | zma00902 | 3 | 0 | 3 | 7 | 0.0204 |
|  |  | Glucosinolate biosynthesis | zma00966 | 2 | 0 | 2 | 3 | 0.0237 |
|  |  | Pentose phosphate pathway | zma00030 | 8 | 0 | 8 | 40 | 0.0267 |
|  |  | Carotenoid biosynthesis | zma00906 | 5 | 1 | 6 | 27 | 0.0327 |
|  |  | Ubiquinone and other terpenoid-quinone biosynthesis | zma00130 | 6 | 0 | 6 | 28 | 0.0384 |
|  |  | Plant-pathogen interaction | zma04626 | 16 | 1 | 17 | 119 | 0.0436 |
|  |  | Indole alkaloid biosynthesis | zma00901 | 2 | 0 | 2 | 4 | 0.0446 |
|  |  | Lysine degradation | zma00310 | 4 | 1 | 5 | 22 | 0.0455 |
| CK12 vs. Inf12  h vs Inf12h |  | Phenylalanine metabolism | zma00360 | 14 | 1 | 15 | 30 | 1.48E-08 |
|  |  | Photosynthesis - antenna proteins | zma00196 | 0 | 11 | 11 | 21 | 7.24E-07 |
|  |  | Isoquinoline alkaloid biosynthesis | zma00950 | 8 | 1 | 9 | 14 | 7.42E-07 |
|  |  | Tyrosine metabolism | zma00350 | 9 | 2 | 11 | 24 | 3.93E-06 |
|  |  | Glutathione metabolism | zma00480 | 18 | 1 | 19 | 68 | 1.04E-05 |
|  |  | Tropane, piperidine and pyridine alkaloid biosynthesis | zma00960 | 7 | 0 | 7 | 12 | 3.39E-05 |
|  |  | Linoleic acid metabolism | zma00591 | 6 | 1 | 7 | 12 | 3.39E-05 |
|  |  | Phenylpropanoid biosynthesis | zma00940 | 23 | 6 | 29 | 142 | 4.73E-05 |
|  |  | Alanine, aspartate and glutamate metabolism | zma00250 | 11 | 2 | 13 | 42 | 8.21E-05 |
|  |  | Monoterpenoid biosynthesis | zma00902 | 4 | 1 | 5 | 7 | 0.0001 |
|  |  | Phenylalanine, tyrosine and tryptophan biosynthesis | zma00400 | 11 | 0 | 11 | 35 | 0.0002 |
|  |  | Diterpenoid biosynthesis | zma00904 | 6 | 1 | 7 | 16 | 0.0003 |
|  |  | Zeatin biosynthesis | zma00908 | 7 | 0 | 7 | 17 | 0.0005 |
|  |  | alpha-Linolenic acid metabolism | zma00592 | 9 | 1 | 10 | 33 | 0.0007 |
|  |  | Flavonoid biosynthesis | zma00941 | 6 | 2 | 8 | 27 | 0.0027 |
|  |  | Arginine biosynthesis | zma00220 | 6 | 1 | 7 | 24 | 0.0054 |
|  |  | Cysteine and methionine metabolism | zma00270 | 12 | 2 | 14 | 73 | 0.0076 |
|  |  | Photosynthesis | zma00195 | 2 | 11 | 13 | 70 | 0.0129 |
|  |  | Valine, leucine and isoleucine biosynthesis | zma00290 | 4 | 1 | 5 | 16 | 0.0134 |
|  |  | Glycolysis / Gluconeogenesis | zma00010 | 16 | 1 | 17 | 102 | 0.0144 |
|  |  | Stilbenoid, diarylheptanoid and gingerol biosynthesis | zma00945 | 4 | 1 | 5 | 17 | 0.0176 |
|  |  | beta-Alanine metabolism | zma00410 | 6 | 1 | 7 | 30 | 0.0193 |
|  |  | MAPK signaling pathway - plant | zma04016 | 14 | 4 | 18 | 115 | 0.0219 |
|  |  | C5-Branched dibasic acid metabolism | zma00660 | 2 | 1 | 3 | 7 | 0.0220 |
|  |  | Glucosinolate biosynthesis | zma00966 | 2 | 0 | 2 | 3 | 0.0251 |
|  |  | Sulfur metabolism | zma00920 | 4 | 2 | 6 | 25 | 0.0260 |
|  |  | Carbon fixation in photosynthetic organisms | zma00710 | 7 | 2 | 9 | 46 | 0.0261 |
|  |  | Valine, leucine and isoleucine degradation | zma00280 | 7 | 0 | 7 | 33 | 0.0317 |
|  |  | Sesquiterpenoid and triterpenoid biosynthesis | zma00909 | 3 | 0 | 3 | 8 | 0.0328 |
|  |  | Thiamine metabolism | zma00730 | 2 | 2 | 4 | 14 | 0.0368 |
|  |  | Pantothenate and CoA biosynthesis | zma00770 | 4 | 1 | 5 | 21 | 0.0423 |
|  |  | Ubiquinone and other terpenoid-quinone biosynthesis | zma00130 | 5 | 1 | 6 | 28 | 0.0434 |
|  |  | Nitrogen metabolism | zma00910 | 3 | 3 | 6 | 28 | 0.0434 |
|  |  | Glycine, serine and threonine metabolism | zma00260 | 8 | 0 | 8 | 43 | 0.0457 |
|  |  | ABC transporters | zma02010 | 3 | 1 | 4 | 15 | 0.0465 |
|  |  | Indole alkaloid biosynthesis | zma00901 | 2 | 0 | 2 | 4 | 0.0471 |

**Supplementary Table 5** Analysis of expression levels of DEGs of various pathways enriched at 3 h, 6 h, and 12 h after *Ustilago maydis* inoculation

| **Pathway** | **Gene ID** | **CK3h** | **CK6h** | **CK12h** | **Inf3h** | **Inf6h** | **Inf12h** | **Description** |
| --- | --- | --- | --- | --- | --- | --- | --- | --- |
| Plant hormone signal transduction | *Zm00001d030028* | -0.965473 | -0.315936 | -1.210882 | 1.011621 | 1.159703 | 0.320967 | myc transcription factor7 |
|  | *Zm00001d043515* | -0.608921 | -0.63817 | -1.343962 | 0.488715 | 1.113536 | 0.988803 | IAA6-auxin-responsive Aux/IAA family member |
|  | *Zm00001d047017* | -0.946519 | -0.554869 | -1.090303 | 0.737092 | 1.381616 | 0.472984 | Putative HLH DNA-binding domain superfamily protein |
|  | *Zm00001d033049* | -0.867649 | -0.549506 | -0.998942 | 1.226964 | 1.201539 | -0.01241 | ZIM motif family protein |
|  | *Zm00001d022017* | -0.366605 | -0.740868 | -0.992826 | 1.581935 | 0.789259 | -0.27089 | Probable indole-3-acetic acid-amido synthetaseGH3.1 |
|  | *Zm00001d018973* | -0.664431 | -0.378401 | -1.179061 | 1.342197 | 0.949602 | -0.06991 | IAA24-auxin-responsive Aux/IAA family member |
|  | *Zm00001d048263* | -1.289952 | -0.728225 | -0.438236 | 0.906763 | 1.180095 | 0.369555 | Putative tify domain/CCT motif transcription factor family protein%3B ZIM motif family protein |
|  | *Zm00001d026308* | -1.261696 | 0.1508057 | -0.082732 | 0.558968 | 0.772118 | -0.13747 | Auxin-responsive protein SAUR71 |
|  | *Zm00001d016924* | -0.605031 | -0.691065 | -0.985495 | 1.325689 | 0.652557 | 0.303345 | ETHYLENE INSENSITIVE 3-like 5 protein |
|  | *Zm00001d027901* | -1.169988 | -0.472407 | -0.596947 | 1.011217 | 1.42165 | -0.19352 | ZIM motif family protein |
|  | *Zm00001d033050* | -0.956969 | -0.705471 | -0.841936 | 1.044687 | 1.307309 | 0.15238 | ZIM motif family protein |
|  | *Zm00001d003903* | -1.070471 | -0.597345 | -0.766985 | 0.905236 | 1.133597 | 0.395969 | Protein TIFY 9 |
|  | *Zm00001d011377* | -0.927594 | 0.1483636 | -1.257208 | 1.047131 | 0.651592 | 0.337716 | Jasmonic acid-amido synthetase JAR1 |
|  | *Zm00001d028574* | -1.355635 | -0.456584 | 0.9276572 | -0.107 | 0.634133 | 0.357431 | Protein phosphatase 2C 37 |
|  | *Zm00001d022139* | -0.586956 | -0.576151 | -0.924679 | 1.232426 | 0.802166 | 0.053195 | Protein TIFY 10B |
|  | *Zm00001d014253* | -0.98421 | -0.348587 | -0.995328 | 0.927014 | 1.311273 | 0.089838 | ZIM motif family protein |
|  | *Zm00001d014682* | -0.904218 | -0.164072 | -0.932007 | 0.539052 | 1.415112 | 0.046133 | SAUR-like auxin-responsive protein family |
|  | *Zm00001d043515* | -0.608921 | -0.63817 | -1.343962 | 0.488715 | 1.113536 | 0.988803 | IAA6-auxin-responsive Aux/IAA family member |
|  | *Zm00001d033049* | -0.867649 | -0.549506 | -0.998942 | 1.226964 | 1.201539 | -0.01241 | ZIM motif family protein |
|  | *Zm00001d022542* | -1.02876 | -0.910538 | -0.403613 | -0.0186 | 1.5371 | 0.824407 | Transcription factor TGA6 |
|  | *Zm00001d038165* | -0.519723 | -0.812088 | -1.205622 | 0.420034 | 0.917995 | 1.199405 | Putative GID1-like gibberellin receptor |
| Glycolysis / Gluconeogenesis | *Zm00001d047663* | -0.67113 | -0.40912 | -1.43817 | 0.360359 | 1.24458 | 0.91348 | Acetate/butyrate--CoA ligase AAE7 peroxisomal |
|  | *Zm00001d038775* | -0.88526 | -0.70307 | -0.70815 | 0.157426 | 0.971707 | 1.16735 | ATP-dependent 6-phosphofructokinase 3 |
|  | *Zm00001d042453* | -1.00968 | -0.64644 | -0.46467 | -0.33928 | 1.180997 | 1.279069 | aldolase1 |
|  | *Zm00001d008651* | -0.72439 | -1.0677 | -0.41613 | -0.07519 | 0.588197 | 1.695205 | Pyruvate decarboxylase isozyme 2 |
|  | *Zm00001d043986* | -0.41454 | -0.81398 | -1.0406 | 0.236224 | 0.986921 | 1.045963 | Pyruvate kinase |
|  | *Zm00001d052494* | -0.78386 | -0.64893 | -0.93942 | 0.099568 | 1.066599 | 1.206049 | Pyruvate kinase |
|  | *Zm00001d025659* | -0.135 | -0.97364 | -0.82896 | 0.216825 | 0.631267 | 1.08951 | ATP-dependent 6-phosphofructokinase 2 |
|  | *Zm00001d042868* | 0.406814 | 0.230636 | -0.48033 | -0.73824 | -0.49125 | 1.072362 | Aldo-keto reductase family 4 member C9 |
|  | *Zm00001d028759* | -0.70477 | -0.69408 | -1.03705 | 0.541815 | 1.033849 | 0.860238 | Pyruvate decarboxylase isozyme 3 |
|  | *Zm00001d012103* | -0.94073 | -0.5923 | -0.46126 | -0.26019 | 1.139514 | 1.114955 | aldolase2 |
|  | *Zm00001d033931* | -0.53932 | -0.64665 | -0.90607 | -0.04612 | 1.047054 | 1.091097 | Alcohol dehydrogenase 1 |
|  | *Zm00001d051001* | -0.32455 | -0.28067 | -1.21886 | 0.100726 | 1.175968 | 0.547395 | Glyceraldehyde-3-phosphate dehydrogenase 3%2Ccytosolic |
|  | *Zm00001d053757* | -0.41343 | -1.04818 | -1.09037 | 0.40001 | 1.165865 | 0.986105 | Galactose mutarotase-like superfamily protein |
|  | *Zm00001d025586* | 0.341708 | -0.56941 | -0.37607 | -0.46711 | -0.39872 | 1.469599 | Galactose mutarotase-like superfamily protein |
|  | *Zm00001d014945* | -0.70845 | -0.69274 | -0.59133 | -0.40567 | 1.298815 | 1.099376 | Lactate/malate dehydrogenase family protein |
|  | *Zm00001d043607* | -0.36237 | -0.46099 | -1.64607 | 0.667443 | 1.268155 | 0.533825 | hexokinase6 |
|  | *Zm00001d031300* | 0.457626 | -0.20219 | -1.47653 | 1.414612 | -0.12104 | -0.07248 | Probable galactinol--sucrose galactosyltransferase1 |
|  | *Zm00001d031303* | 0.40452 | -0.14249 | -1.61877 | 1.202664 | 0.25198 | -0.09791 | Probable galactinol--sucrose galactosyltransferase1 |
|  | *Zm00001d023941* | -1.10701 | -0.46444 | -0.94061 | 0.400263 | 0.984358 | 1.127434 | Carbohydrate transporter/ sugar porter |
|  | *Zm00001d028230* | -1.16351 | -0.65446 | -0.945 | 0.631224 | 1.235198 | 0.896545 | Sugar transport protein 13 |
|  | *Zm00001d042337* | -1.03458 | -0.53964 | -0.54289 | -0.36744 | 0.827634 | 1.656916 | Nucleotide-diphospho-sugar transferase familyprotein |
| Photosynthesis | *Zm00001d003767* | 1.1078349 | 1.1767163 | 0.2399471 | 0.700354 | -0.02072 | -1.20109 | Photosystem I subunit O |
|  | *Zm00001d018797* | 1.0378303 | 1.1687331 | 0.3875032 | 0.650939 | -0.1632 | -1.09104 | photosystem I reaction center6 |
|  | *Zm00001d041819* | 1.0475084 | 1.2046019 | 0.5539549 | 0.45628 | -0.25921 | -0.95238 | photosystem I N subunit1 |
|  | *Zm00001d050403* | 0.8415854 | 1.3500623 | -0.200594 | 0.724356 | 0.530665 | -1.4987 | Chlorophyll a-b binding protein 4 |
|  | *Zm00001d021784* | 1.1627639 | 0.7170677 | -0.840939 | 1.018832 | 0.347 | -1.48162 | Chlorophyll a-b binding protein of LHCII type III |
|  | *Zm00001d006587* | 1.1781938 | 1.1165636 | -0.192453 | 0.831649 | 0.228796 | -1.4945 | Chlorophyll a-b binding protein CP29.1chloroplastic |
|  | *Zm00001d001857* | 1.018527 | 0.8760912 | -0.2446 | 0.832891 | 0.551679 | -1.55775 | light harvesting complex photosystem II subunit 6 |
|  | *Zm00001d005996* | 0.7562224 | 1.7532468 | 0.2786316 | 0.200049 | -0.14299 | -1.10567 | Photosystem I reaction center subunit V |
|  | *Zm00001d006663* | 1.172326 | 1.0311574 | -0.088474 | 0.897288 | 0.258355 | -1.3102 | light harvesting complex A1 |
|  | *Zm00001d007267* | 1.1450275 | 1.052003 | -0.427742 | 0.891492 | 0.19662 | -1.26389 | - |
|  | *Zm00001d013146* | 1.0890033 | 0.8073167 | -0.238865 | 0.951956 | 0.105655 | -1.60676 | Photosystem I reaction center subunit IIIchloroplastic |
|  | *Zm00001d019518* | 0.954694 | 1.5633722 | 0.133998 | 0.406106 | 0.087302 | -1.30211 | Photosystem I reaction center subunit IV A |
|  | *Zm00001d020877* | 0.8970383 | 1.2006427 | 0.636972 | 0.116878 | -0.34587 | -0.5167 | Photosystem I reaction center subunit Vchloroplastic |
|  | *Zm00001d021435* | 0.8464146 | 0.9241822 | -0.799605 | 0.845143 | 0.65585 | -1.61683 | - |
|  | *Zm00001d021763* | 1.06167 | 1.5400238 | 0.1554394 | 0.406537 | -0.00341 | -1.12405 | photosystem II subunit29 |
|  | *Zm00001d021906* | 1.1199781 | 0.6985863 | -0.435378 | 1.251829 | 0.160237 | -1.52933 | Chlorophyll a-b binding protein |
|  | *Zm00001d026599* | 1.0211644 | 0.8224158 | 0.2588983 | 0.811633 | 0.23124 | -0.98201 | - |
|  | *Zm00001d032051* | 1.7628777 | -0.214023 | -0.254759 | 0.851871 | -0.03842 | -1.28708 | light harvesting complex photosystem II subunit 6 |
|  | *Zm00001d038984* | 1.0232888 | 1.3132083 | 0.2509245 | 0.533532 | 0.13019 | -1.32415 | photosystem I H subunit1 |
|  | *Zm00001d046786* | 1.0165354 | 1.2499709 | -0.695161 | 0.763036 | 0.430437 | -1.46759 | Photosystem I chlorophyll a/b-binding protein 3-1chloroplastic |
|  | *Zm00001d048998* | 1.1066506 | 1.1241336 | 0.1959487 | 0.585128 | -0.00504 | -0.8938 | Chlorophyll a-b binding protein CP26 chloroplastic |

**Supplementary Table 6** Log_2_ fold change in genes differentially expressed between control (CK) and inoculated samples (Inf) at various time points after *U. maydis* inoculation

| **Pathway** | **Gene ID** | **CK3h vs Inf3h** | **CK6h vs Inf6h** | **CK12h vs Inf12h** |  |
| --- | --- | --- | --- | --- | --- |
| Plant hormone signal transduction | *Zm00001d016924* | 2.460559592 | 2.165619868 | 3.740914176 |  |
|  | *Zm00001d011377* | 1.488482813 | 0.240604441 | 1.03011438 |  |
| Glycolysis / Gluconeogenesis | *Zm00001d031303* | 0.756177412 | 0.326728815 | 1.822277164 |  |
|  | *Zm00001d028230* | 3.602637082 | 3.658189099 | 3.527733904 |  |
|  | *Zm00001d042337* | 4.563554951 | 2.127444579 | 2.919997837 |  |
|  | *Zm00001d023941* | 2.17487582 | 1.687136907 | 2.360903161 |  |
|  | *Zm00001d042453* | 0.663374406 | 1.854793508 | 1.601076131 |  |
|  | *Zm00001d043607* | 0.661004192 | 1.066118916 | 1.337778428 |  |
|  | *Zm00001d012103* | 0.67087183 | 1.522881723 | 1.381810048 |  |
|  | *Zm00001d038775* | 0.699458075 | 1.170122334 | 1.120933713 |  |
| Photosynthesis | *Zm00001d003767* | -0.306104555 | -1.072352812 | -1.243464285 |  |
|  | *Zm00001d018797* | -0.305881063 | -1.174894014 | -1.228253642 |  |
|  | *Zm00001d041819* | -0.553134831 | -1.276046064 | -1.268706584 |  |
|  | *Zm00001d038984* | -0.281077615 | -0.902741368 | -1.070831739 |  |
|  | *Zm00001d050403* | -0.104365169 | -0.860903839 | -1.229137713 |  |
|  | *Zm00001d021784* | -0.274777369 | -1.131367531 | -2.334959396 |  |
